# Development of versatile non-homologous end joining-based knock-in module for genome editing

**DOI:** 10.1038/s41598-017-18911-9

**Published:** 2018-01-12

**Authors:** Shun Sawatsubashi, Yudai Joko, Seiji Fukumoto, Toshio Matsumoto, Shigeo S. Sugano

**Affiliations:** 1 Department of Molecular Endocrinology, Fujii Memorial Institute of Medical Sciences, Institute of Advanced Medical Sciences, Tokushima University, Tokushima, Japan; 2 Ritsumeikan Global Innovation Research Organization, Ritsumeikan University, Shiga, Japan; 3 PRESTO, Japan Science and Technology Agency, Saitama, Japan

## Abstract

CRISPR/Cas9-based genome editing has dramatically accelerated genome engineering. An important aspect of genome engineering is efficient knock-in technology. For improved knock-in efficiency, the non-homologous end joining (NHEJ) repair pathway has been used over the homology-dependent repair pathway, but there remains a need to reduce the complexity of the preparation of donor vectors. We developed the versatile NHEJ-based knock-in module for genome editing (VIKING). Using the consensus sequence of the time-honored pUC vector to cut donor vectors, any vector with a pUC backbone could be used as the donor vector without customization. Conditions required to minimize random integration rates of the donor vector were also investigated. We attempted to isolate null lines of the *VDR* gene in human HaCaT keratinocytes using knock-in/knock-out with a selection marker cassette, and found 75% of clones isolated were successfully knocked-in. Although HaCaT cells have hypotetraploid genome composition, the results suggest multiple clones have *VDR* null phenotypes. VIKING modules enabled highly efficient knock-in of any vectors harboring pUC vectors. Users now can insert various existing vectors into an arbitrary locus in the genome. VIKING will contribute to low-cost genome engineering.

## Introduction

Genome engineering changed dramatically with the invention of the CRISPR/Cas9-based genome-editing system and subsequent improvements^1,2^. Ribonucleoprotein complex of *Streptococcus pyogenes* Cas9 protein with a single guide RNA (gRNA) specifically binds to the NGG sequence (called the protospacer adjacent motif, PAM), with the sequence directed by gRNA, and induces double strand breaks (DSB) in genomic DNA^3^. The programmable feature of the Cas9–gRNA complex can be used to modify the genome.

One of the most important applications of genome editing is to improve “knock-in” technology, which is the technology for inserting exogenous DNA fragments into arbitrary sites of the target genome^1^. The development of knock-in technology began with the “gene targeting technique”^4^. The gene targeting technique harnesses the endogenous homology directed repair (HDR) pathway to insert DNA fragment precisely into the target site of the genome^4^. However, the gene targeting technique remains generally inefficient. This is because the activity of the HDR pathway is commonly low in cells. When a DSB is induced by an artificial nuclease, there are three possible DNA repair pathways: the canonical non-homologous end joining (c-NHEJ) pathway; the alternative NHEJ pathway (also called the microhomology-dependent pathway); and the HDR pathway^5^. As the c-NHEJ pathway occurs with high frequency, using the c-NHEJ pathway is favorable to achieve highly efficient knock-in.

There are several methods that use the c-NHEJ pathway to knock-in^6–12^. These methods simultaneously induce DSB in both genomic and donor vectors in living cells. Ligation using the c-NHEJ pathway between the linearized donor vector and the cleaved genome occurs *in vivo*. Several studies have reported highly efficient knock-in using c-NHEJ. For example, knock-in with an efficiency of 30% or more was reported in plants and zeblafish^9,12^. CRISPaint technology efficiently enables tag-encoding DNA fragments to be added into endogenous genes^11^. In addition to the c-NHEJ pathway, the microhomology-dependent pathway has been used for knock-in^13,14^. The technology uses the microhomology sequence in both the target genome and the donor vector, and the donor vector can be seamlessly inserted into the site with microhomology at the target genome.

Even though c-NHEJ-based knock-in is highly efficient, one of the problems with these techniques is the complexity associated with the preparation of donor vectors, and it is necessary to redesign and construct new donor vectors. In the early development methods^8,9^, the target sequence of the artificial nuclease for cutting the knock-in donor vector must be designed each time. Recent study shows the practical protocols to simplify the construction of donor vectors^15^. However, there are still rooms for technical improvement to use various donor vector.

Another problem of c-NHEJ-based knock-in technology is random integration. It is possible that transfected vectors would integrate into an unwanted locus in the genome. However, to the best of our knowledge, systematic analysis of random integration into the genome has not been conducted.

The biologically active form of vitamin D [1α, 25-dihydroxyvitamin D (1,25(OH)2D)] has multiple roles in the body, including regulation of intestinal calcium and phosphate absorption, maintenance of bone mineral density and modulation of cell proliferation and differentiation in the skin^16–18^. Genomic actions of 1,25(OH)_2_D are accomplished by its binding to vitamin D receptor (VDR), which forms a heterodimer with retinoid X receptor alpha and subsequently binds to vitamin D response elements (VDRE) to either enhance or repress transcription of various target genes. The human vitamin D 24-hydroxylase gene (*CYP24A1*) is one of these target genes and is strongly upregulated by 1,25(OH)_2_D which binds to two VDRE in the proximal promoter region^19^. Interestingly, loss of function mutations of *VDR* result in several phenotypes resembling aging, including early alopecia, thickened skin, and keratinocyte abnormalities in the epidermis in humans and mice^20,21^. Although the VDR is a well-known modulator of calcium homeostasis in the intestines, kidney and bone, the functions of VDR in keratinocytes remain unclear.

In the current study, we developed the versatile NHEJ-based knock-in modules for genome editing (VIKING), which does not require reconstruction of donor vectors depending on the target sequence on the genome. As a module for cleaving donor vectors, a consensus sequence of pUC19^22^ was selected as the target. Because a number of vectors are derived from the pUC vector, VIKING enables various existing vectors to be used as donors. Additionally, this study sought to overcome the random integration problem by optimizing the introduction ratio of the donor cleaving vector and the target cleaving vector. The results suggest that it is possible to minimize the labor required to prepare donor vectors and achieve highly efficient knock-in using the VIKING method in mammalian cell lines.

## Results

### Design of versatile knock-in module using c-NHEJ-based knock-in

**Figure 1:**
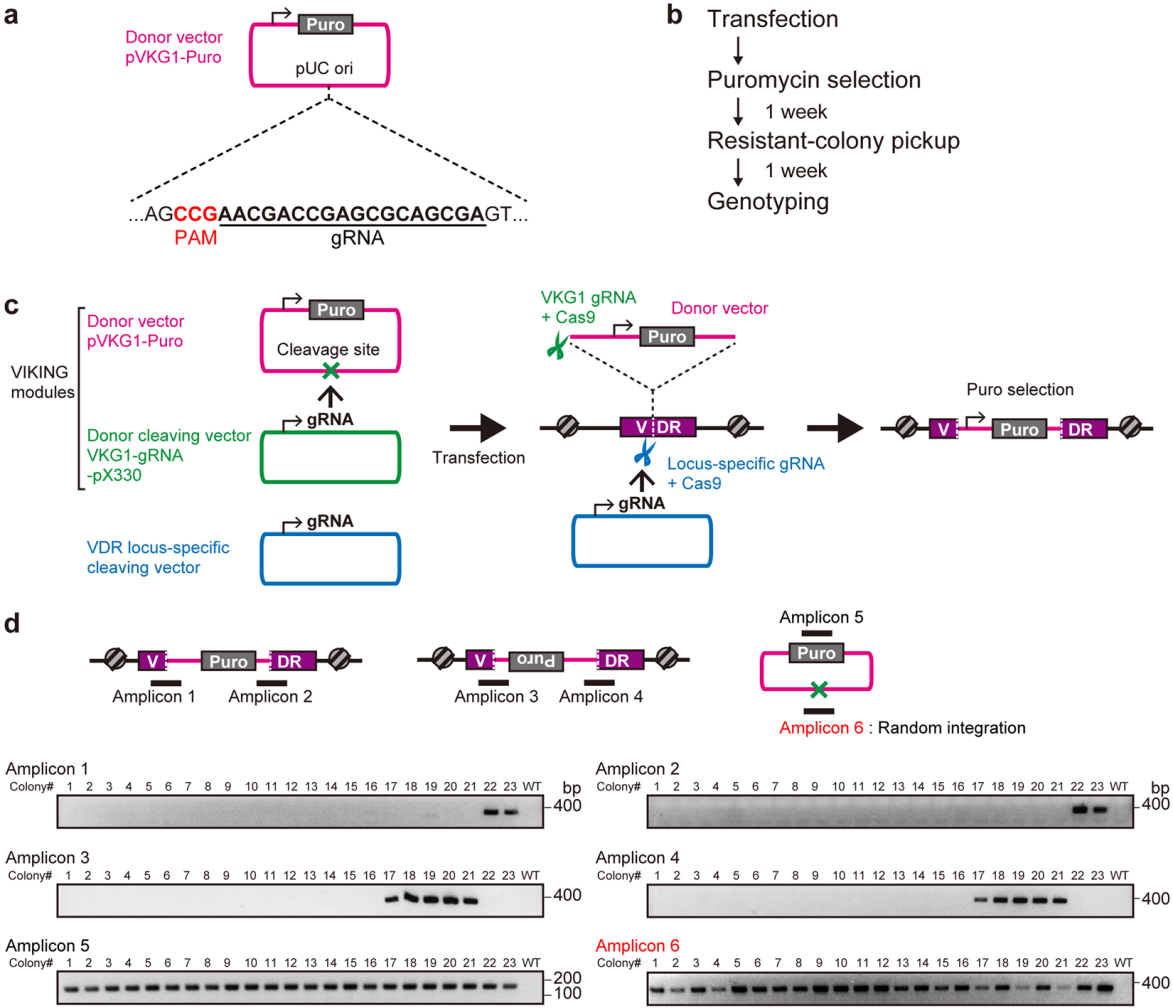
Design and assessment of versatile NHEJ-based knock-in modules for genome editing (VIKING). (**a**) The gRNA sequence used for donor plasmid cleavage. The gRNA sequence is not present in human and mouse genomes but can target the donor plasmids, which have a pUC backbone. (**b**) The experimental flow of non-homologous end joining (NHEJ)-based knock-in. (**c**) Schematic of knock-in vector sets and methods used to establish knock-in HaCaT cell lines. The system comprises three vectors: a donor vector, having an antibiotic-resistance gene as a selection marker (magenta); a donor plasmid cleavage vector (green); and a vector that cleaves a specific site in the genome (blue), in this case, an exon of the *vitamin D receptor* (*VDR*). (**d**) Knock-in efficiency under transfection conditions of the molar ratio of the vectors (1:1:1). Schematic PCR products are written (Amplicon 1–6). If Amplicon 1 and Amplicon 2 were amplified, the insertion of the donor vector is defined as “the forward direction.” On the other hand, if Amplicon 3 and Amplicon 4 were amplified, it is defined as “the reverse direction.” If the knock-in events occur by random integrations, Amplicon 6 should be amplified.

We first designed NHEJ-based knock-in system using a CRISPR/Cas9 expression plasmid (derived from pX330^23^) containing gRNA targeting *VDR* (the human *vitamin D receptor*gene), a donor vector containing a puromycin-resistance gene (*PURO*), and a donor cleaving vector (Fig. 1a–c). To cleave the donor plasmid, a sequence commonly existing in the backbone of the pUC19 vector was selected. The sequence TCGCTGCGCTCGGTCGTT was chosen as the donor cleaving site because the sequence is one of the fewest that harbors off-target sites in human and mouse genomes (Supplementary Table S1). The gRNA sequence length was designed 18 nucleotides, because the truncated gRNA shows low off-target effects^24^. The sequence was defined as VKG1 (Fig. 1a). A circular plasmid harboring the VKG1 sequence and *PURO* cassette was used as the donor vector (pVKG1–Puro). As a vector for cleaving donors, the VKG1 gRNA sequence was cloned into the highly efficient CRISPR/Cas9 vector pX330 (designated VKG1-gRNA-pX330). The pX330 vector does not contain the VKG1 sequence and would not be cleaved by the donor cleaving vector itself. HaCaT keratinocyte cells were simultaneously transfected with a *VDR* locus-specific CRISPR/Cas9 vector, pVKG1–Puro, and VKG1-gRNA-pX330. The stable resistant colonies were treated with puromycin (0.3 µg/mL) for 14 days. PCR genotyping was used to confirm whether insertion of the donor vector into the *VDR* locus or random integration occurred (Fig. 1b,c). The genotyping was also performed considering two patterns of knock-in occurring at the *VDR* locus. Namely, the knock-in insertion in one direction (Fig. 1d; Amplicon 1 and Amplicon 2) and that in the other one (Fig. 1d; Amplicon 3 and Amplicon 4). In the resistant clones, Amplicon 6, containing the VKG1 sequence, which should be cut when the donor vector was cleaved, was also detected by PCR (Fig. 1d; Amplicon 6). Accordingly, the percentage of random integration free clone was 0%. These results suggested that random integration of the donor vector had occurred without cleavage of the VKG1 sequence and conferred puromycin resistance.

### Assessment of random integrations suggested that the molar ratio of the donor vector and the target cleaving vector is a critical factor

**Figure 2:**
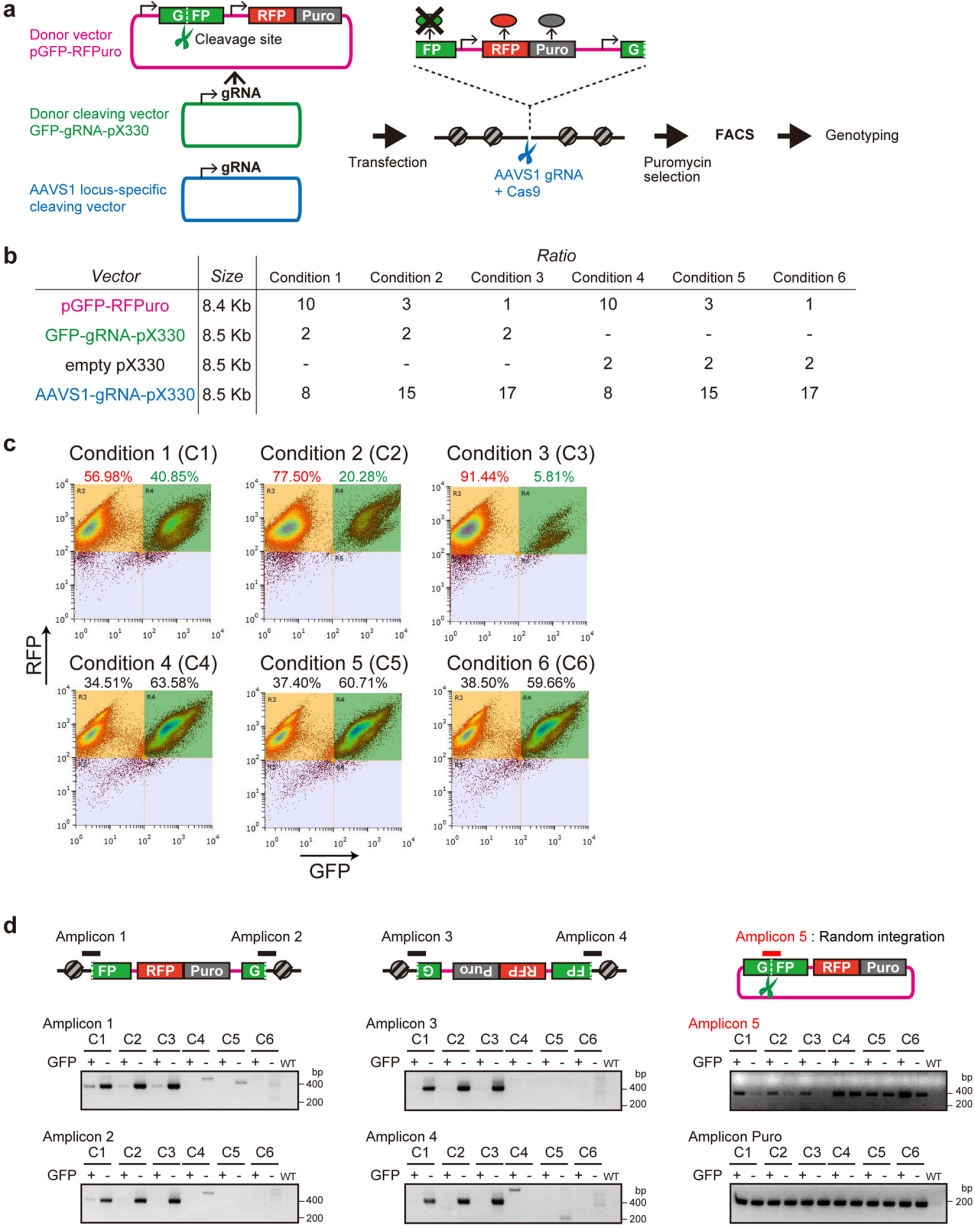
Optimization of the molar ratio of the vectors to reduce unwanted random integration. (**a**) Experimental design to measure the random integration of the donor plasmid and the knock-in events at the target site. To detect random integrations, a plasmid with a (green fluorescent protein) *GFP* expression cassette and a cassette expressing *RFP-2A-Puro* was designed as the donor (magenta). As a donor cleaving vector (green), a CRISPR/Cas9 vector expressing gRNA targeting the *GFP* sequence was designed. The *hAAVS1* locus was used as the target locus (blue). (**b**) Experimental conditions to assess random integration rate. The donor vector, the donor cleaving vector, and the target cleaving vector were transfected simultaneously into HEK293F cells at various molar ratios (Conditions 4–6 are negative controls). (**c**) FACS analysis of cells by fluorescent intensity of GFP and red fluorescent protein (RFP). If the insertion shows random integration, it was expected that GFP fluorescence would be detected. The fewer donor vectors used, the fewer random integrations occurred. (**d**) Genotyping PCR to confirm knock-in events in RFP-positive cells sorted by FACS. GFP+ shows a cell population expressing both GFP and RFP. GFP− shows a cell population expressing only RFP. C1–C6 shows Condition 1 to Condition 6 in (B). Amplicon 5 indicates random integration.

The frequency of random integration of the donor vector has not been analyzed in detail in previous studies^6–12^. Therefore, we developed an assay system to assess the conditions under which the donor vector was randomly inserted into the genome without being cleaved (Fig. 2a). The system was assessed in the HEK293F cells, which are widely used in life science. The conditions assessed using this cell line would be valuable to the various users of mammalian cells. As a donor cleaving vector, a CRISPR/Cas9 vector expressing gRNA targeting the green fluorescent protein (GFP) sequence was constructed. The human adeno-associated virus integration site 1 (*hAAVS1*) locus was used as the target locus^25^.

To detect random integration, a plasmid with a *GFP* expression cassette and a cassette expressing red fluorescent protein (RFP)−2A-Puro was designed as the donor. If random integration occurred in this system, the puro-resistant cells would show both GFP fluorescence and RFP fluorescence. Cells with a successful knock-in event without any random integration could be detected by the loss of GFP fluorescence. To determine the optimal conditions for knock-in, the three vectors (the donor vector, the donor cleaving vector, and the target cleaving vector) were transfected simultaneously into HEK293F cells at various molar ratios (Fig. 2b). The transfectants were treated with puromycin for 14 days and subjected to analysis by cell sorting (Fig. 2c). If cleavage of the donor vector by CRISPR/Cas9 had occurred, the percentage of cells exhibiting GFP fluorescence should decrease. On the other hand, the GFP expression cassette in the donor vector occupies 20% length of the vector. Therefore, if the donor vector were randomly cut and integrated into the host genome, 20% of the transfectants would show only RFP signal without any genome editing events. It was observed in our experiment (Fig. 2c, Condition 4-6).

We observed that the fewer donor vectors used, the fewer random integrations occurred. The population with random integration events decreased to 5.81% when the ratio of the target cleaving vector to the donor vector was 17:1 (Fig. 2b,c Condition 1–3).

Genomic DNA from a mixture of sorted cells was extracted from a GFP-negative/RFP-positive population and a GFP-positive/RFP-positive population. PCR genotyping was performed to check that the knock-in event had occurred at the target locus (Fig. 2d). As expected, in GFP-negative/RFP-positive cells, PCR products resulting from the knock-in were obtained (Fig. 2d; Amplicon 1–4). Random integration events were not detected in these cell groups (Fig. 2d; Amplicon 5). In GFP-positive population, it is possible that the donor vectors may have been integrated into both the target and random locus in the genome (Fig. 2d, Amplicon 1–4, Condition 1). The results of these experiments suggested that reducing the number of molecules of the donor vector is important to reduce unwanted random integration.

### c-NHEJ-based knock-in occurred when the circular donor vector was cleaved after transfection

To analyze the knock-in reaction using the c-NHEJ pathway, we attempted knock-in using linear double-stranded DNA as the donor. A CRISPR/Cas9 expression plasmid containing gRNA targeting *VDR* and a linearized donor vector containing a puromycin-resistance gene (*PURO*) were simultaneously introduced into HaCaT cells with the optimized ratio (Supplementary Fig. S1). HaCaT cells showing resistance to puromycin could be isolated in only 23 colonies out of 5 × 10^6^ transfected cells. The knocked-in event in the target loci was not observed in the isolated cells, on the contrary to the condition using circular plasmids (Supplementary Fig. S1b). These results suggested that intracellular cleavage of the donor vector is important for efficient knock-in using the c-NHEJ pathway.

### Highly efficient knock-in using VIKING modules in HaCaT cells

**Figure 3:**
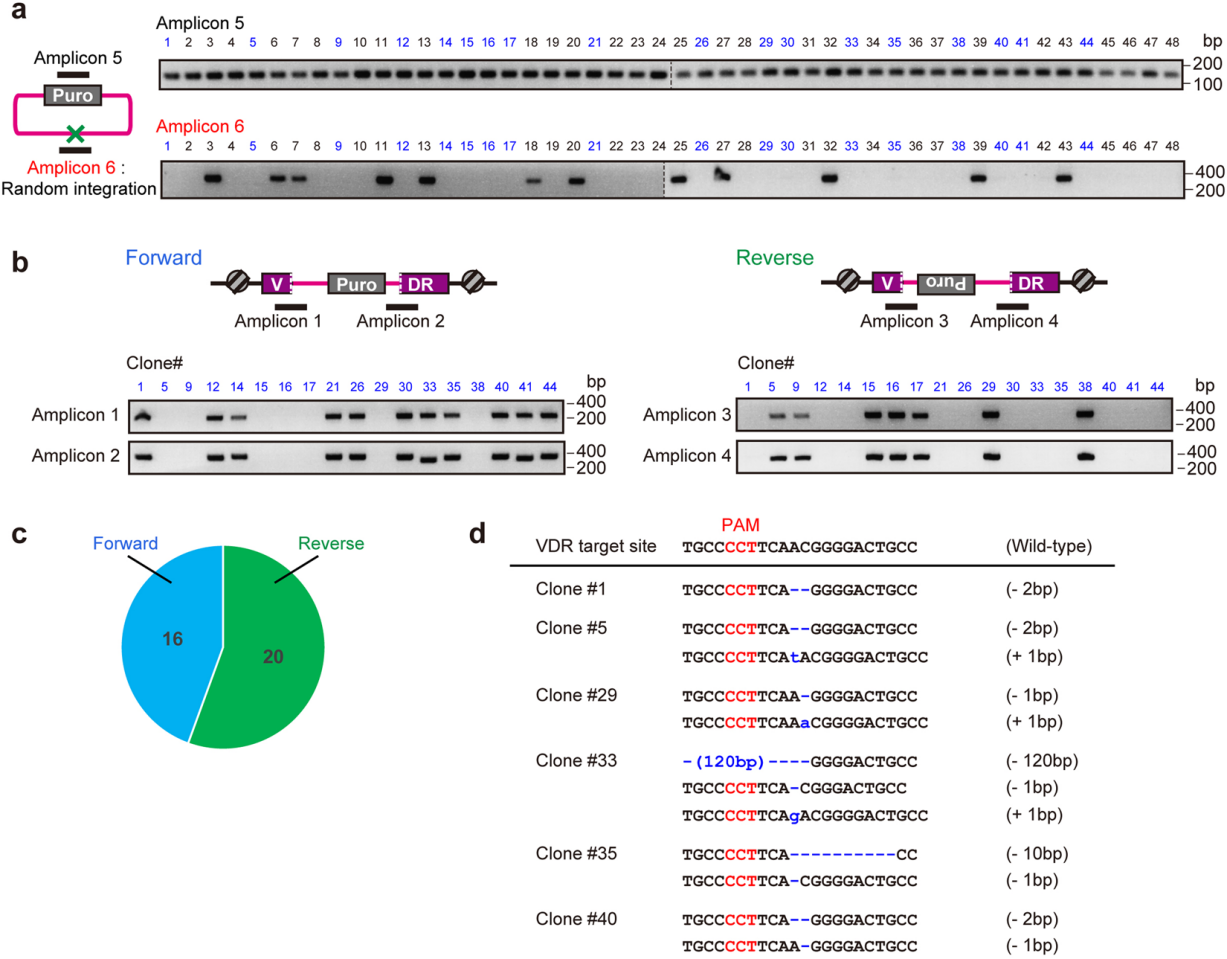
Knock-in experiment at the *VDR* locus in hypotetraploid HaCaT cells under the optimal conditions. (**a**) Assessment of random integration of puro-resistant cells. Knock-in cells without the targeted random integration in 36/48 clones were established. (**c**) Assessment of directions of integrated donor vectors. Schematic PCR products are written (Amplicon 1–6). If Amplicon 1 and Amplicon 2 were amplified, the insertion of the donor vector is defined as “the forward direction.” On the other hand, if Amplicon 3 and Amplicon 4 were amplified, it is defined as “the reverse direction.” (**c**) Ratio of directions of vectors that integrated into the target locus in knock-in cell lines. (**d**) Representative sequences of the *VDR* locus in the knock-in cell lines.

To investigate whether the optimized molar ratio of vectors for transfection is applicable to other cell lines, we examined whether a donor containing the *PURO* cassette could be knocked-in at the *VDR* locus in HaCaT cells. The *VDR* cleaving vector (VDR–pX330), a donor cleaving vector (VKG1-gRNA-pX330), and a donor vector (pVKG1–PURO) were simultaneously introduced into HaCaT cells at a ratio of 17:2:1 (Fig. 1c; conditions modified). After treatment with puromycin, the puro-selected cell colonies were cloned and subjected to PCR genotyping. The puro-resistant cassette was amplified in all cell lines (Fig. 3a; Amplicon 5), but unwanted random integration was observed only in 12 out of 48 samples (Fig. 3a; Amplicon 6). Therefore, it was inferred that random integration events did not occur in 75% of the cells selected (36/48). Analyses of additional clones showed 53/70 (75.7%) clones had no random integration (No amplification of Amplicon 6, Fig. 3; Supplementary Tables S2, S3). The donor was correctly inserted into the target locus in multiple clones (Fig. 3b). Direction of the knock-in events were not so biased (16 samples in one direction and 20 samples in the other direction) in 36 samples in which random integrations were not observed (Fig. 3c).

Using direct sequencing analysis, the presence or absence of indels at donor insertion sites were investigated in 18 clones (Fig. 3b, Supplementary Fig. S2, Supplementary Table S2). Over the half of the clones (10/18 clones) had no error-prone mutations at the integration sites; moreover, 28 out of 36 junctions analyzed were the sequences with precise knock-in. Southern blotting analyses of representative clones corresponded to the results of PCR analyses (Supplementary Fig. S3). The optimized condition would enable correctly to insert the donor into the target locus and reduced unwanted integration of donors. Sequencing analysis of the additional knock-in clones also confirmed that most of the junctions are precise integration; 59/81 junctions had no indel (Supplementary Table S3). Deletions were frequently found in the PAM distal position. The apparent sequence bias was not observed. In addition, sequences *VDR* locus of the chromosomes without knock-in events were also analyzed in the 18 representative clones (Supplementary Table S4, Fig. 3d). The direct sequencing analyses suggested that some of the clones with knock-in event can be *VDR null* mutants.

### Functional characteristics of VDR knock-in/knock-out in HaCaT cell lines

**Figure 4:**
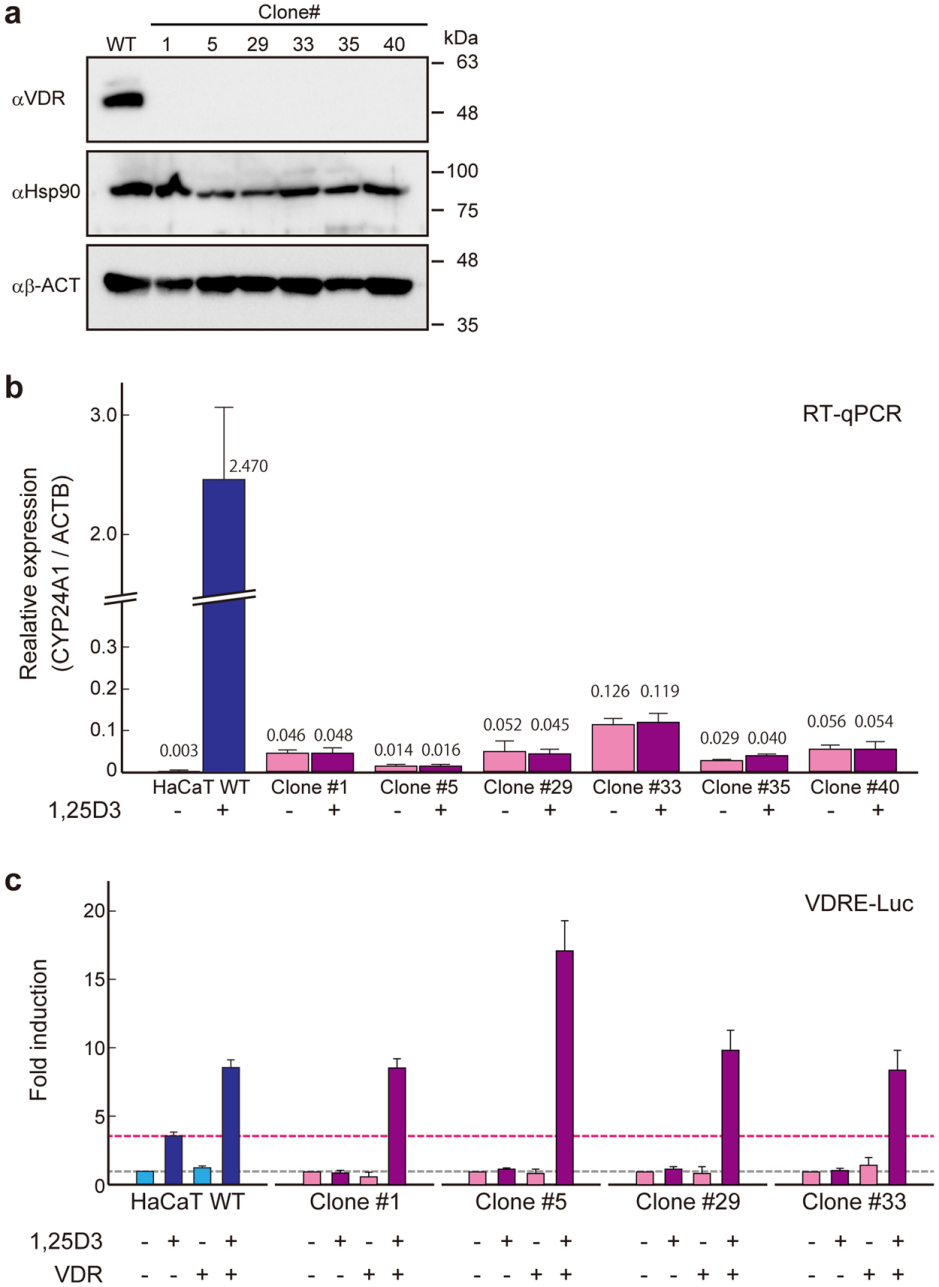
Confirmation of loss-of-function of the *VDR* gene in the knocked-in cell lines. (**a**) Immunoblot analyses of isolated clones. αHsp90 and αβ-ACT were used as loading controls. (**b**) RT-qPCR analysis of *CYP24A1* of the isolated cells with or without treatment with 1α,25-dihydroxyvitamin D3 (1,25D3). (**c**) Promoter-reporter assay using vitamin D response element (VDRE). When endogenous *VDR* was functional, VDRE would respond to 1,25D3 treatment. The exogenous *VDR* expression plasmid (VDR+) was transfected for the complementation assay.

HaCaT is a non-transformed keratinocyte cell line with a hypotetraploid karyotype and has four copies of chromosome 12 containing the *VDR* gene locus. Because almost all the isolated knock-in cells had two or more different mutations at *VDR* locus (Supplementary Table S4), it is possible that the isolated clones were not *VDR* null. Therefore, we analyzed *VDR* gene activity of the representative knock-in lines obtained. No VDR protein was detected in these cell lines using immunoblot analysis (Fig. 4a).

The human vitamin D 24-hydroxylase gene (*CYP24A1*) is strongly upregulated by active vitamin D3 (1,25(OH)2D3) through two VDRE in the proximal promoter region^19^. As expected, induction of *CYP24A1* mRNA expression was not observed in knock-in lines compared with wild-type HaCaT cell lines (Fig. 4b). Finally, transactivation via VDRE at the human *CYP24A1* promoter was examined in each HaCaT cell line using the luciferase reporter assay. In wild-type HaCaT cells, activation of VDRE reporter was observed with the addition of 1,25(OH)2D3, whereas no response was observed in the knock-in cell lines (Fig. 4c; lane VDR−). When the *VDR* gene expression plasmid was additionally introduced, activation of VDRE reporter by 1,25(OH)2D3 occurred in these knock-in lines. With transfection of the exogenous *VDR* gene, the insensitivity of the knock-in cell line to 1,25(OH)2D3 was rescued (Fig. 4c; lane VDR+). The results suggested that these clones are loss-of-function mutants of the *VDR* gene.

### Assessment of Versatility of VIKING modules

In the VIKING method, any vectors harboring VKG1 sequence can be used as donor vectors. To check versatility of the VIKING method, the knock-in experiment to *VDR* locus using both pVKG1-PURO and another donor vector, pVKG1-BSD, was conducted using HaCaT cells (Supplementary Fig. S4). pVKG1-BSD harbors a blasticidin resistant cassette. The knock-in lines were isolated by co-selection using puromycin and blasticidin. Among five independent colonies survived in the selection, two knocked-in lines were successfully isolated (Supplementary Fig. S4a). These clones harbored the donor vectors of both pVKG1-PURO and pVKG1-BSD (Supplementary Fig. S4a). The cleavage sites of knock-in lines showed the error-prone indels (Supplementary Fig. S4b). The isolated knock-in clones showed complete loss of VDR protein expression (Supplementary Fig. S4c).

Since the knock-in strategy using the VIKING method can be applied to any locus, we also tried to get knock-in/knock-out HaCaT cell lines in another gene locus, *Hairless* (*HR*) (Supplementary Fig. S5). Using pVKG1-PURO as a donor vector, the knock-in colonies in *HR* locus were successfully selected by puromycin. Three out of five puro-resistant clones had successful knock-in (Supplementary Fig. S5a). Seamless knock-in of the donor vector to the target *HR* locus were detected by direct sequencing analysis in one clone (Supplementary Fig. S5b,c). In addition, the rest of chromosome which had no donor knock-in event showed indels at the gRNA target site of *HR* (Supplementary Fig. S5d). Immunoblot analysis of the selected clones showed the reduction of HR protein level (Supplementary Fig. S5e). Collectively, it can be concluded that the VIKING method can be applied to various locus using vectors harboring VKG1 sequence as a donor.

## Discussion

**Figure 5:**
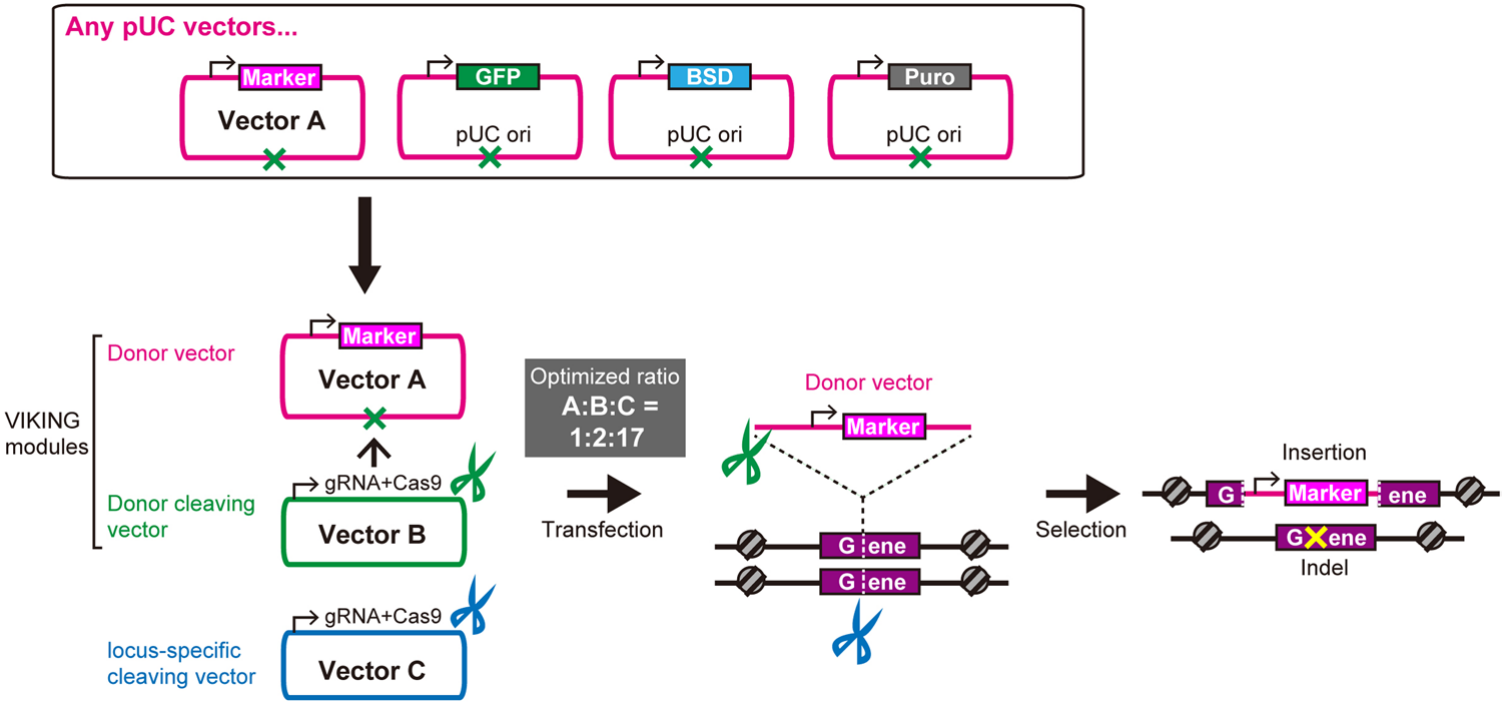
Schematic representation of non-homologous end joining (NHEJ)-based knock-in using versatile NHEJ-based knock-in modules for genome editing (VIKING). Step one: select the donor plasmid, which harbors the VIKING–gRNA1 sequence (Vector A). Any vector with a pUC backbone could be used as the donor vector without any customization from publicly available resources. Step two: construct the vector for cleaving the target genome (Vector C). Step three: simultaneously transfect the three vectors. Step 4: select cell lines harboring successful knock-in.

In this study, we sought to determine the optimal conditions for knock-in using the c-NHEJ pathway and developed a highly versatile knock-in module (the VIKING module) (Fig. 5). Previous studies have reported that the c-NHEJ pathway can be used for knock-in more efficiently than the HDR pathway; however, optimization of the balance between random integrations and precise knock-in are not reported.

In the current study, we determined two important conditions for efficient knock-in using the c-NHEJ pathway. First, cleavage of the donor vector should occur within cells (Supplementary Fig. S1). This is suggested by the understanding that c-NHEJ initiation factors such as KU70 and KU80 would be attached to the 5′ end or 3′ end of target DNA fragments in response to DSB in cells and facilitate ligation of each end^26^. It was also confirmed in recent work using CRISPR/Cas9^27^. Second, transfection using fewer donor vectors was favorable to avoid random integrations (Figs 1–3). This might be because there are too many donor plasmids in cells. The CRISPR/Cas9 system as the vector for cleaving the donor vector would not be able to digest all the donor vectors, which leads to random integration of donor plasmids in the genome. The study suggests that random integration would occur not only in a donor vector but also in a target cleaving vector or a donor cleaving vector. Therefore, transfection of Cas9–gRNA ribonucleoprotein, which harbors the VKG1 gRNA sequence and target genome sequence, with a donor vector would help avoid unwanted random integration of the transfected plasmids^14^.

A potential problem of the VIKING method is that it is not able to control the number of chromosomes where knock-in occurs. Although HaCaT cells are known to have a hypotetraploid genome^28^, only one of the four homologous chromosomes was shown to be knocked-in in almost all the clones isolated by one selection marker. In this study, we obtained two of the four homologous chromosomes harbored knock-in using two different selection markers (Supplementary Fig. S4). To achieve knock-in of all chromosomes simultaneously, it is thought that additional protocols, such as control of the cell cycle, would be required.

In this study, the *VDR* gene was disrupted in HaCaT cells, a model of human keratinocytes that maintains full epidermal differentiation capacity. The sequencing analyses shows indels induced by CRISPR/Cas9 in *VDR* locus (Fig. 3d, Supplementary Table S4) would lead the disruption of *VDR* by frameshifts. The *VDR* gene is known to function in epidermal tissue; however, this has not been confirmed in HaCaT cells. The results of *CYP24A1* mRNA expression analysis and the reporter assay (Fig. 4) are consistent with previous findings^29^. HaCaT cells are useful as a model system for vitamin D signaling in keratinocytes. Although HaCaT cells are hypotetraploid cells, it is relatively easy to establish *VDR* null strains using the VIKING system.

The greatest advantage of the VIKING module (using a donor cleaving vector and a donor vector harboring the VKG1 sequence), is that any donor vectors harboring the VKG1 sequence can be used without any additional vector modifications (Fig. 5). The VKG1 sequence exists in vectors such as pcDNA3, pBluescript II, pENTR, and pUC. Because theses vectors have been used as the backbones of most existing vectors, it is possible to insert most existing vectors into an arbitrary locus using the VIKING method. In recent years, a systematic mechanism to share vectors (http://www.addgene.org/) has been developed^30^. Various resistance markers such as blasticidin, neomycin, puromycin, hygromycin, and zeocin tolerance are now ready for use in knock-in because pUC-based vectors harboring such resistance cassettes have been deposited (https://www.addgene.org/vector-database/). Many of these vectors are ready to use as donor vectors with the VIKING system (Fig. 5).

In the VIKING method, c-NHEJ based repair has been occurred, which would possibly cause small indel at target sites (Fig. 3d). One of the important application of knock-in is to insert the GFP reporter in downstream of the promoter of interest, which need a precisely seamless integration of foreign DNA into the target. The effectiveness of the application of knock-in has been confirmed in previous studies, including CRISPaint methods^11,13^. It is necessary to try to reduce frequency of unwanted indels at target sites in the VIKING method in the future study. Furthermore, in principle, the VIKING method could also be applied to knock-in mice^31^. It is possible that microinjection of VIKING modules to zygote would accelerate to make knock-in mice. Isolation of knock-in lines without any selection marker would be challenging. Because there is a reduction in labor associated with knock-in using the VIKING system, the system will further accelerate the generation of knock-in cell lines and functional genetics.

## Methods

### VIKING modules construction

To make donor vectors, the EF1a-Puro-polyA cassette was PCR-amplified using pCDH vectors as the templates and sub-cloned into the pENTR-based vector. To make the donor cleaving modules, we cloned the VKG1 sequence using annealed oligo CAACGTCGCTGCGCTCGGTCGTT and AAACACTTCTGACAACGATCGGC into *Bbs* I-digested pX330 (Addgene; #42230). For genome editing of the *VDR* locus and *hAAVS1*, annealed oligos comprising the sequences of each gene (*VDR*, 5′-CACCGATGCGGCAGTCCCCGTTGA-3′, 5′-AAACTCAACGGGGACTGCCGCATC-3′, *hAAVS1*, 5′-CACCGAGAGCCACATTAACCGGCCC-3′, 5′-AAACGGGCCGGTTAATGTGGCTCTC-3′) were cloned into pX330. The eGFP sequence was inserted into the pCDH-CMV-MCS-EF1-RFP + Puro vector (CD516B-2; System Biosciences, Palo Alto, CA). Primers used in this study are listed in Supplementary Table S5. pX330-U6-Chimeric_BB-CBh-hSpCas9 was a gift from Feng Zhang (Addgene; #42230).

### Plasmids

For mammalian cell expression, cDNA of the 5′-terminally FLAG-His6-tagged hVDR was inserted in pcDNA3 (Life Technologies, Carlsbad, CA)^32^. For the reporter assay, the CYP24A1 promoter region was amplified by PCR and inserted into the pGL4.27 vector (Promega, Madison, WI). Detailed information is available in the Supplementary Table S5.

### Cell culture, transfection, and selection

The non-tumorigenic immortalized human keratinocyte HaCaT cell line was obtained from Dr. Yoko Yamamoto (University of Tokyo, Tokyo, Japan). HaCaT or human embryonic kidney T cell F (HEK293F) cells were cultured in Dulbecco’s modified Eagle’s medium (DMEM) (043-30085; Wako, Osaka, Japan) supplemented with 10% fetal bovine serum (S1780-500; Biowest, Nuaillé, France) and 1 U penicillin–streptomycin (161-23181; Wako). HaCaT cells were suspended in Opti-MEM (11058-021; Life Technologies) and electroporated with 15 µg DNA in the VIKING modules and pX330 plasmids using a pulse generator (CUY21EDITII; Bex, Tokyo, Japan). Electroporated cells were seeded into 100-mm dishes and pre-cultured in DMEM without antibiotics for 24 h. Transduced HaCaT cells were selected following puromycin treatment (0.3 µg/mL) for 14 days to isolate clonal colonies. HEK293F cells were transfected with the indicated VIKING modules using TurboFect transfection reagent (R0531; Thermo Fisher Scientific, Waltham, MA). Cells were seeded into 100-mm dishes and pre-cultured in DMEM without antibiotics for 24 h. Transduced HEK293F cells were selected following puromycin treatment (1 µg/mL) for 14 days and subjected to cell sorting using a cell sorter (S3e; Bio-Rad, Hercules, CA).

### PCR genotyping and direct sequencing analysis

For genotyping, PCR amplification from genomic DNA of transfected cell lines was conducted using GoTaq master mix (M7123; Promega) according to the manufacturer’s protocol. Each PCR product was directly sequenced using internal primers. Primer sets are described in the Supplementary Table S5.

### RNA preparation and RT-qPCR

To estimate the expression level of *CYP24A1*, wild-type or knock-in HaCaT cells were treated with 1,25(OH)2D3 (10^−7^ M calcitriol, FC09794; Carbosynth, Compton, UK) for 24 h. Total RNA was isolated from HaCaT cells using RNAiso Plus (9109; Takara, Tokyo, Japan), and cDNA was synthesized using PrimeScript RT master mix (RR036A; Takara) according to the manufacturer’s protocol. Quantitative PCR analysis was performed using a LightCycler 96 (Roche. Basel, Switzerland) with FastStart Essential DNA Green Master (04673492001; Roche). Results were calculated as mean ± S.D. from at least three independent experiments. Primer sets are described in the Supplementary Table S5.

### Reporter assay

To assess the vitamin D response of the isolated knock-in lines, HaCaT cells were transfected with the indicated plasmids (0.5 µg VDRE–Luc and 0.5 µg hVDR) using Transficient DNA transfection reagent (WU1003; MBL International, Woburn, MA) into 12-well plates. The total amount of cDNA was adjusted by supplementing with an empty vector up to 1.0 µg. Cells were treated with 1,25(OH)2D3 for 24 h. Luciferase activity was determined using GloMax Discover (Promega) and the Dual-Luciferase assay system (E1980; Promega). As a reference plasmid to normalize the transfection efficiency, 80 ng pRL-TK plasmid (E2241; Promega) was co-transfected in all experiments. Results were calculated as mean ± S.D. from at least three independent experiments^33^.

### Immunoblot analysis

Immunoblot was conducted following methods described previously^32^. Briefly, each HaCaT cell extract (from approximately 10^7^ cells) was prepared with RIPA lysis buffer (50 mM HEPES, pH 7.9, 150 mM NaCl, 1% NP-40, 0.5% Na-deoxycolate, 0.1% SDS) supplemented with protease inhibitor cocktail (P8340; Sigma-Aldrich, St. Louis, MI) and sonicated using a Bioruptor sonicator (UCD-250; Cosmo Bio, Tokyo, Japan). The extracts were subjected to SDS-PAGE and blotting using standard protocols. In case of analysis of Hairless protein, immunoprecipitation using UltraLink Immobilized Protein G (#53125; Thermo Fisher Scientific) with anti-HR (ab37540; Abcam, Cambridge, UK) antibody were conducted before SDS-PAGE analysis. Detailed protocol of the immunoprecipitation was previously described^32^. Immunoblotting with anti-VDR (12550; Cell Signaling Technology, Danvers, MA), anti-Hsp90 (sc-7947; Santa Cruz Biotechnology, Dallas, TX), anti-β-actin (sc-47778; Santa Cruz Biotechnology), or anti-HR(AF5708; R&D systems, Minneapolis, MN) antibodies was performed as described in the Supplemental Material and measured using the ChemiDoc Touch imaging system (Bio-Rad).

### Southern blotting

Southern blotting was conducted by the protocol previously described^34^. Briefly, the probe was generated by the PCR amplification using primers “5′-GCTGTTTCCTGGCAGCTCTG-3′” and “5′- TTGCGCCGTCCCGTCAAGTC-3′” to get 997 bp DNA fragment of *VDR*, and labelled by the DIG DNA labeling and detection kit following manufactures protocol (#11093657910; Roche). Genomic DNAs were purified from HaCaT cell lines using basic phenol extraction method. The genomic DNAs were digested with PstI, NotI, or BsrGI, respectively. Digested DNA fragments were separated by agarose gel electrophoresis and transferred to nylon membranes (#11209299001; Roche). The membrane was incubated with the DIG-labeled DNA probe and the chemiluminescent signals were detected by following manufactures protocol with the ChemiDoc Touch imaging system (Bio-Rad).

## Electronic supplementary material

Supplementary information
