# Supplementary material for: Development of versatile non-homologous end joining-based knock-in module for genome editing
Source: Sci Rep. 2018 Jan 12;8:593. doi: 10.1038/s41598-017-18911-9 (PMC5766535; doi:10.1038/s41598-017-18911-9)

Supplementary Dataset for

Development of versatile non-homologous end joining-based knock-in module for genome editing

Shun Sawatsubashi[1*], Yudai Joko[1], Seiji Fukumoto[1], Toshio Matsumoto[1], Shigeo S. Sugano[2,3*]

1 Department of Molecular Endocrinology, Fujii Memorial Institute of Medical Sciences, Institute of Advanced Medical Sciences, Tokushima University, Tokushima, Japan

2 Ritsumeikan Global Innovation Research Organization, Ritsumeikan University, Shiga, Japan

3 PRESTO, Japan Science and Technology Agency, Japan

* Corresponding authors

This PDF files includes :

Supplementary Figs. S1 to S5

Supplementary Tables S1 to S5

Supplementary Information (including the uncropped gel and blot source data)

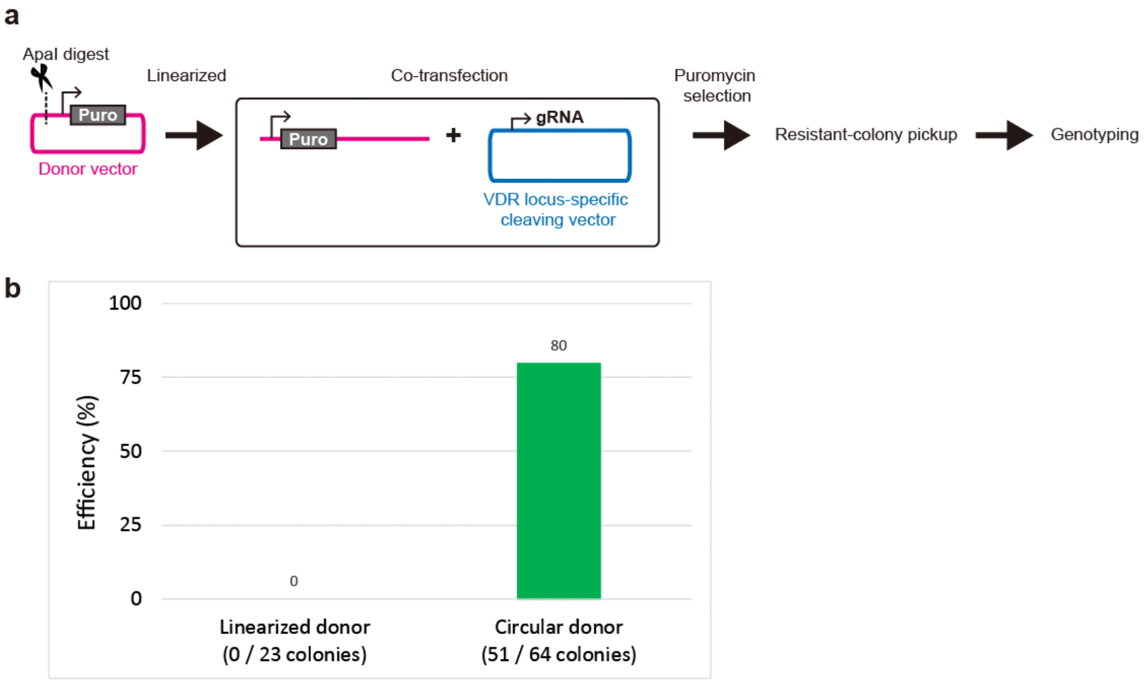

Supplementary Fig. S1 Knock-in efficiency using a pre-linearized donor vector. (a) Schematic representation of knock-in strategy mediated a pre-linearized donor vector. The *PURO*-cassette vector was digested with ApaI enzyme. (b) The efficiency of knock-in events using linearized or circular donor vector with the condition optimized in Fig. 2.

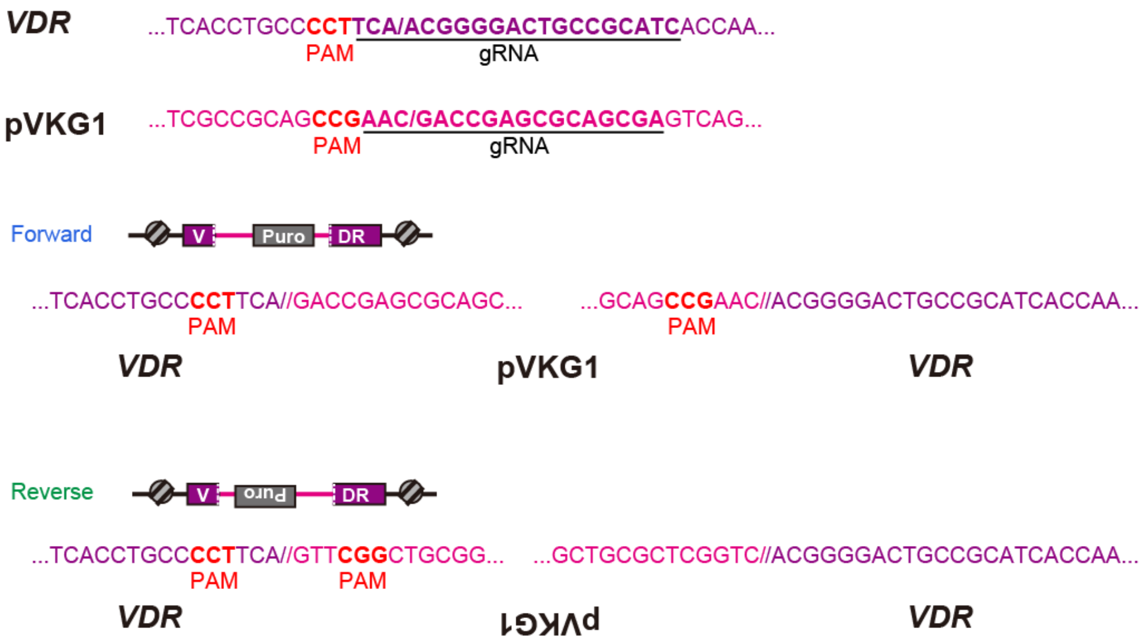

Supplementary Fig. S2 Detailed sequence of the knock-in junctions.

(Upper) The target sequence for cleaving pVKG1-PURO vector (the donor vector) and that for cleaving the *VDR* in the genome. The exact target sites of gRNA were underlined. The PAM sequences were colored in red and shown in bold. (Lower) The predicted sequence of knock-in products, in which the cleaved donor vector was precisely integrated into the target site. The two possible directions of insertion (forward and reverse) were shown. The cleavage site by Cas9 were shown in slash.

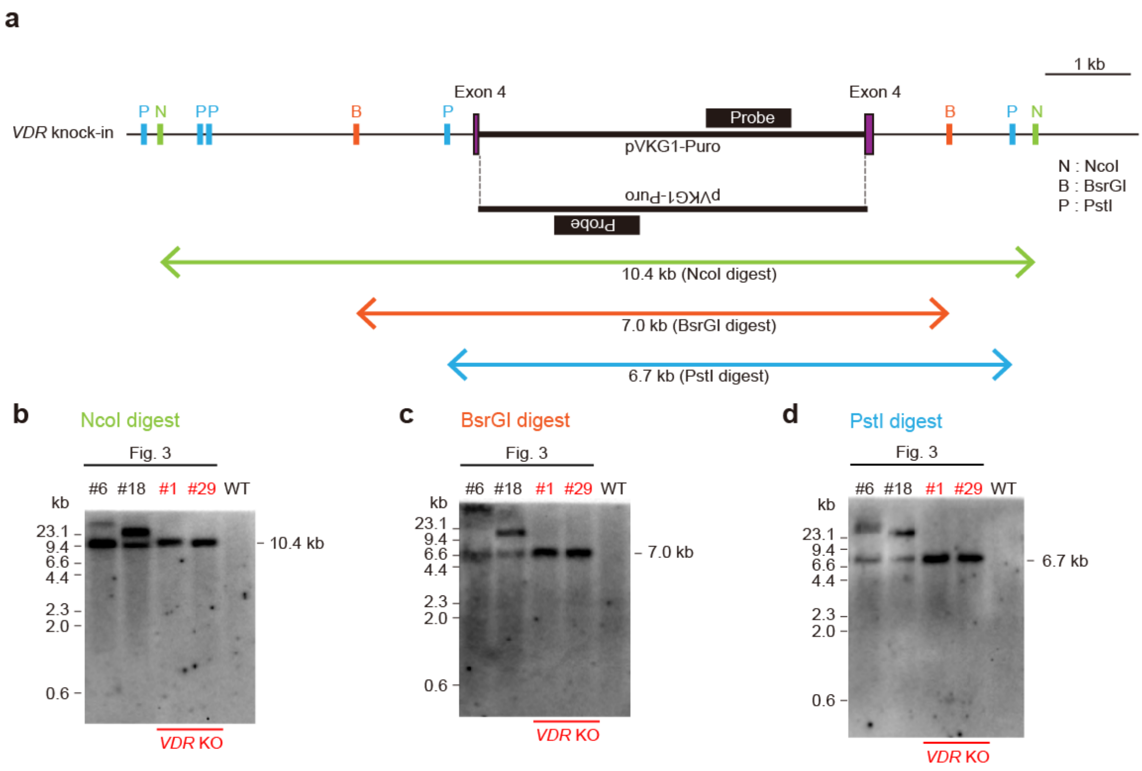

Supplementary Fig. S3 Southern blot analyses of the genomes of clones which are described in Fig. 3a.

(a) Schematic image of the experimental design of southern blotting. The specific probe for a donor vector pVKG1-Puro was shown as “Probe.” Expected sizes of the genome fragment digested by three restriction enzymes were shown. (b-d) Southern blot analysis of the genome of knock-in clones digested by NcoI (b), BsrGI (c), and PstI (d). #6, #18 were clones categorized as “random integrations” (Amplicon 6 positive). #1 and #29, which were red-colored, were clones categorized as “no random integrations” (Amplicon 6 negative). WT shows the analysis of the HaCaT cells without transfection. Clone numbers are corresponding to those in Figure 3.

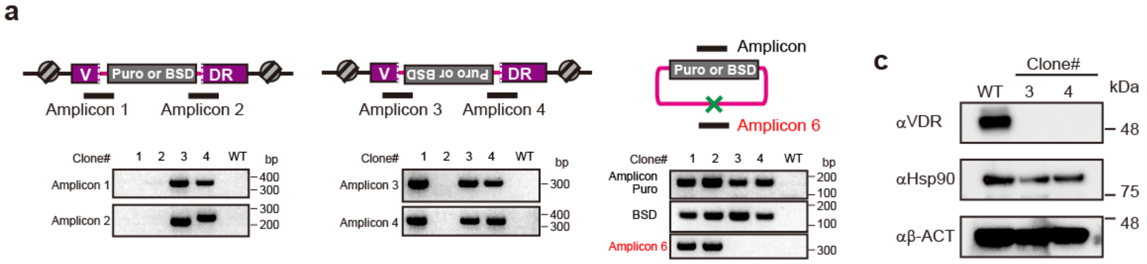

b

Cleavage sites	Clone #1	indel size	Clone #3	indel size	Clone #4	indel size
Amplicon 1	No integration		...TGCCCCTTCA/GACCGAGCGC...	(0 / 0)	...TGCCCCTTCA/GACCGAGCGC...	(0 / 0)
Amplicon 2	No integration		...--(30bp)--/-------CTG...	(-30 / -7)	...GCAGCCGAAC/-CGGGGACTG...	(0 / -1)
Amplicon 3	...TGCCCCTTCA/GTTCGGCTGC...	(0 / 0)	...TGCCCCTTCA/GTTCGGCTGC...	(0 / 0)	...TGCCCCTTCAA/GTTCGGCTGC...	(+1 / 0)
Amplicon 4	...GCGCTCGGTC/-CGGGGACTG...<br>...GCGCTCGGTC/--GGGGACTG...	(0 / -1)<br>(0 / -2)	...GCGCTCGGTC/ACGGGGACTG...	(0 / 0)	...GCGCTCGGTC/ACGGGGACTG...	(0 / 0)
VDR	...TGCCCCTTCA/-CGGGGACTG...	(-1)	...TGCCCCTTCA/AACGGGGACTG...	(+1)	....TGCCCCTTCA/-CGGGGACTG...	(-1)

Supplementary Fig. S4 Simultaneous knock-in of two donor vectors using VIKING method.

(a-c) Simultaneous knock-in of two different vectors. The pVKG1-PURO and pVKG1-BSD were used as donor vectors. (a) Confirmation of directions of integrated donor vectors by PCR analysis. Schematic PCR products were written (Amplicon 1-6). Schematic PCR products are written (Amplicon 1-6). If Amplicon 1 and Amplicon 2 were amplified, the insertion of the donor vector is defined as “the forward direction.” On the other hand, if Amplicon 3 and Amplicon 4 were amplified, it is defined as “the reverse direction.” (b) Direct sequencing analysis of the junctions of the knock-in sites. (c) Immunoblot analyses of isolated clones. αHsp90 and αβ-ACT were used as loading controls.

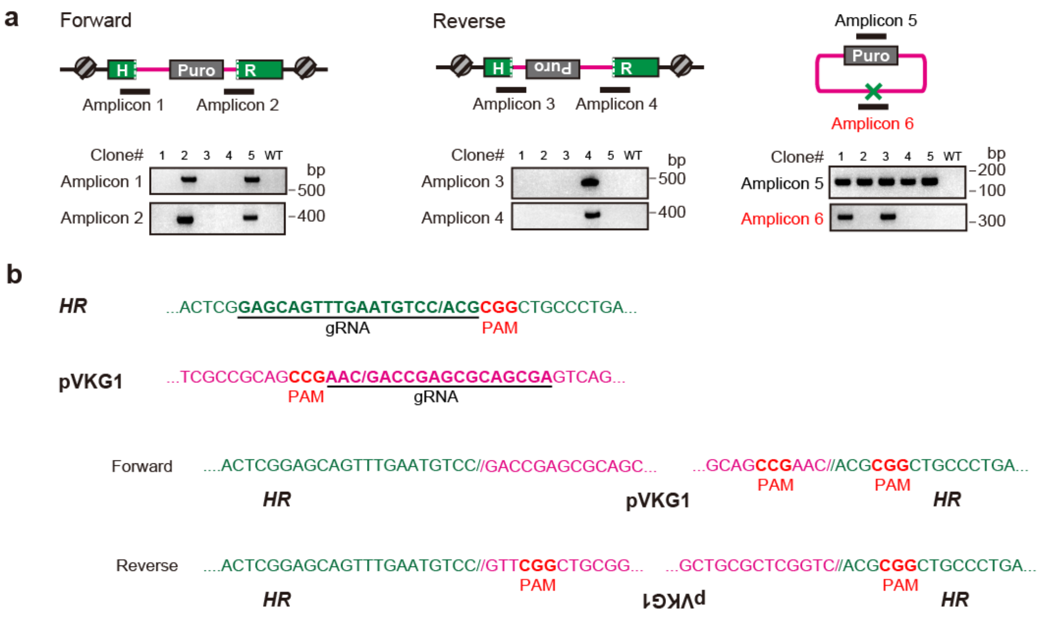

c

Clone	Direction (Forward, Reverse)	Upstream (HR genomic DNA)	Upstream (Plasmid DNA)	indel size	Downstream (HR genomic DNA)	Downstream (Plasmid DNA)	indel size
**Predicted sequence (Forward)**		ACTCGGAGCAGTTTGAATGTCC/	/GACCGAGCGCAGCGAGTCAGTG		TCTCCTCGACCTCAGGGCAGCCGCGT/	/GTTCGGCTGCGGCGAGCGGTAT	
**Predicted sequence (Reverse)**		ACTCGGAGCAGTTTGAATGTCC/	/GTTCGGCTGCGGCGAGCGGTAT		TCTCCTCGACCTCAGGGCAGCCGCGT/	/GACCGAGCGCAGCGAGTCAGTG	
#2	Forward	ACTCGGAGCAGTTTGAATGTCC/	/GACCGAGCGCAGCGAGTCAGTG	0/0	TCTCCTCGACCTCAGGGCAGCCGCG-/	/-------------AGCGGTAT	-1/-14
#4	Reverse	ACTCGGAGCAGTTTGAATGTCC/	/GTTCGGCTGCGGCGAGCGGTAT	0/0	TCTCCTCGACCTCAGGGCAGCCGCGT/	/GGACCGAGCGCAGCGAGTCAGTG	0/+1
#5	Forward	ACTCGGAGCAGTTTGAATGTCC/	/GACCGAGCGCAGCGAGTCAGTG	0/0	TCTCCTCGACCTCAGGGCAGCCGCGT/	/GTTCGGCTGCGGCGAGCGGTAT	0/0

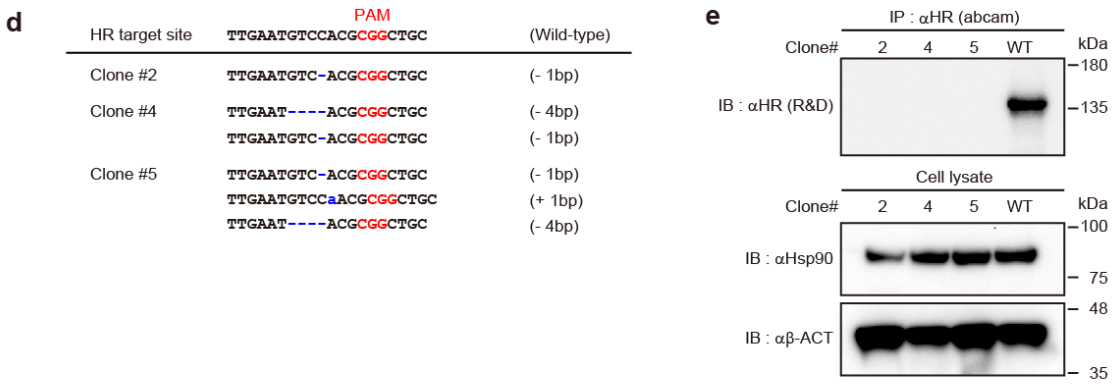

Supplementary Fig. S5 Isolation of knock-in/knock-out clones in *HR* locus using HaCaT cells.

(a) Confirmation of directions of integrated donor vectors in *HR* locus by PCR analysis. Schematic PCR products were written (Amplicon 1-6). (b) (Upper) Sequences for cleaving pVKG1-PURO vector (the donor vector) and *HR* locus in the genome. The exact target sites of gRNA were underlined. The PAM sequences were colored in red

and shown in bold. (Lower) The predicted sequence of knock-in products, in which the cleaved donor vector was precisely integrated into the target site. The two possible directions of insertion (forward and reverse) were shown. The cleavage site by Cas9 wasshown in slash. (c) The summary of direct sequencing analyses of knock-in clones. The junction sites of the knock-in clones were shown. (d) Sequences of the *HR* locus in the genome of knock-in cell lines analyzed by direct sequencing of PCR product. (e) Immunoblot analyses of isolated clones. Immunoprecipitated products using αHR (abcam) antibody (Upper) and cell lysates (Lower) were analyzed using αHR (R&D), αHsp90 and αβ-ACT antibodies.

Supplementary Table S1 Design of VKG1 sequence using CRISPRdirect

Using Homo sapiens genome data (hg19), potential off targets of all the gRNA target sequences in the pUC19 vector were analyzed by CRISPRdirect[35].

Column1-2: the position of nucleotide and direction of the strand in pUC19. The sequence of pUC19 was obtained from Addgene Plasmid #50005.

Column4: the target sequences of gRNA with PAM. Column5-7: Number of off targets which match the gRNA target sequence of PAM proximal 20 mer (hit_20mer), 12mer (hit_12 mer), and 8mer (hit_8mer).

The gRNA target with fewest off target was shown in yellow and its 18 nt trancated version was defined as VKG1.

Ref 35. Naito Y, Hino K, Bono H, Ui-Tei K. CRISPRdirect: software for designing CRISPR/Cas guide RNA with reduced off-target sites. *Bioinformatics* **31**, 1120-1123 (2015).

# start	end	strand	sequence	hit_20mer	hit_12mer	hit_8mer		
2642	2664	-	ccgaacgaccgagcgcagcgagt	0	2	82	VKG1	18nt
1287	1309	+	ttacttctgacaacgatcggagg	0	0	83		
1398	1420	-	ccaaacgacgagcgtgacaccac	0	0	108		
25	47	-	ccccgcgcgttggccgattcatt	0	2	168		
672	694	-	ccgaaacgcgcgagacgaaaggg	0	1	172		
1651	1673	+	tcgtagttatctacacgacgggg	0	1	194		
568	590	-	ccgctgacgcgccctgacgggct	0	3	195		
2195	2217	+	aagacgatagttaccggataagg	0	2	228		
2372	2394	+	cggaacaggagagcgcacgaggg	0	3	232		
27	49	-	ccgcgcgttggccgattcattaa	0	0	250		
2509	2531	-	ccagcaacgcggcctttttacgg	0	1	258		
327	349	-	cctggcgttacccaacttaatcg	0	2	272		
158	180	-	ccggctcgtatgttgtgtggaat	0	1	280		
1124	1146	-	ccgtattgacgccgggcaagagc	0	0	281		
1645	1667	-	cccgtatcgtagttatctacacg	0	1	282		
2358	2380	+	gtaagcggcagggtcggaacagg	0	0	287		
1646	1668	-	ccgtatcgtagttatctacacga	0	2	295		
1650	1672	+	atcgtagttatctacacgacggg	0	2	295		
15	37	+	aaccgcctctccccgcgcgttgg	0	2	299		
1004	1026	+	acgagtgggttacatcgaactgg	0	2	302		
2600	2622	-	ccgtattaccgcctttgagtgag	0	1	307		
1116	1138	+	gtattatcccgtattgacgccgg	0	0	309		
26	48	-	cccgcgcgttggccgattcatta	0	3	323		
481	503	+	gcggtatttcacaccgcatatgg	0	0	330		
5	27	-	ccaatacgcaaaccgcctctccc	0	0	331		
2064	2086	+	cttctagtgtagccgtagttagg	0	1	341		
2168	2190	+	taagtcgtgtcttaccgggttgg	0	0	351		
735	757	+	ataatggtttcttagacgtcagg	0	3	356		
298	320	+	tcgttttacaacgtcgtgactgg	0	0	361		
467	489	-	ccttacgcatctgtgcggtattt	0	0	369		
2371	2393	+	tcggaacaggagagcgcacgagg	0	7	401		
269	291	-	ccgggtaccgagctcgaattcac	0	4	402		
398	420	-	ccgcaccgatcgcccttcccaac	0	2	417		
1284	1306	+	aacttacttctgacaacgatcgg	0	1	443		
326	348	-	ccctggcgttacccaacttaatc	0	2	447		
2571	2593	-	cctgcgttatcccctgattctgt	0	2	450		
1352	1374	+	tgtaactcgccttgatcgttggg	0	2	457		
1351	1373	+	atgtaactcgccttgatcgttgg	0	1	461		
268	290	-	cccgggtaccgagctcgaattca	0	5	469		
2430	2452	-	cctgtcgggtttcgccacctctg	0	0	486		
2158	2180	-	ccagtggcgataagtcgtgtctt	0	0	491		
45	67	+	attaatgcagctggcacgacagg	0	3	496		
374	396	+	cagctggcgtaatagcgaagagg	0	3	497		
1361	1383	-	ccttgatcgttgggaaccggagc	0	7	503		
990	1012	+	gatcagttgggtgcacgagtggg	0	2	512		
220	242	-	ccatgattacgccaagcttgcat	0	1	527		
2464	2486	+	gatttttgtgatgctcgtcaggg	0	4	528		
397	419	-	cccgcaccgatcgcccttcccaa	0	1	542		
431	453	-	cctgaatggcgaatggcgcctga	0	2	552		
4	26	-	cccaatacgcaaaccgcctctcc	0	4	554		
1203	1225	+	acagaaaagcatcttacggatgg	0	2	555		
1649	1671	+	tatcgtagttatctacacgacgg	0	4	561		
2275	2297	-	cctacaccgaactgagataccta	0	2	563		
2355	2377	-	ccggtaagcggcagggtcggaac	0	7	566		
1642	1664	-	cctcccgtatcgtagttatctac	0	0	569		
1117	1139	+	tattatcccgtattgacgccggg	0	0	575		
57	79	+	ggcacgacaggtttcccgactgg	0	3	575		
276	298	-	ccgagctcgaattcactggccgt	0	2	587		
2465	2487	+	atttttgtgatgctcgtcagggg	0	2	587		
1641	1663	-	ccctcccgtatcgtagttatcta	0	0	592		
1854	1876	-	ccttaacgtgagttttcgttcca	0	4	594		
2347	2369	+	acaggtatccggtaagcggcagg	0	4	597		
919	941	-	cctttttgcggcattttgcctt	0	1	618		
299	321	+	cgttttacaacgtcgtgactggg	0	0	646		
2466	2488	+	tttttgtgatgctcgtcaggggg	0	2	651		
2343	2365	+	gcggacaggtatccggtaagcgg	0	1	659		
363	385	-	ccccctttcgccagctggcgtaa	0	3	665		
2220	2242	+	cagcggtcgggctgaacgggggg	0	6	688		
2463	2485	+	cgatttttgtgatgctcgtcagg	0	1	696		
267	289	-	ccccgggtaccgagctcgaattc	0	2	701		
989	1011	+	agatcagttgggtgcacgagtgg	0	6	703		
1588	1610	-	ccggtgagcgtgggtctcgcggt	0	4	710		
365	387	-	ccctttcgccagctggcgtaata	0	7	722		
762	784	+	acttttcggggaaatgtgcgcgg	0	5	735		
1061	1083	-	ccccgaagaacgttttccaatga	0	0	737		
579	601	-	ccctgacgggcttgtctgctccc	0	3	748		
580	602	-	cctgacgggcttgtctgctcccg	0	1	756		
366	388	-	cctttcgccagctggcgtaatag	0	9	757		
1853	1875	-	cccttaacgtgagttttcgttcc	0	2	769		

Supplementary Table S2 Direct sequencing analysis of Knock-in cell lines

The sequences of PCR amplicon 1 - 4 in Fig.3B, which were analyzed by direct sequencing, were shown. The predicted sequences with forward and reverse direction were shown in colored sequences.
PAM sequences were shown with red and bold. The indel were colored with red. The predicted sequeces show the sequences with precise knocked-in (see also Supplementary Fig. S2).

Clone	Direction (Forward, Reverse)	Upstream (VDR genomic DNA)	Upstream (Plasmid DNA)	indel size	Downstream (VDR genomic DNA)	Downstream (Plasmid DNA)	indel size	Amplicon 6
**Predicted sequence (Forward)**		CATGAAGCGGAAGGCACTATTCACCTGCC**CCT**TCA/	/GACCGAGCGCAGCGAGTCAGTG		GTCGGTTGTCCTTGGTGATGCGGCAGTCCCCGT/	/GTT**CGG**CTGCGGCGAGCGGTATCAGCTCACTCAAAG		
**Predicted sequence (Reverse)**		CATGAAGCGGAAGGCACTATTCACCTGCC**CCT**TCA/	/GTT**CGG**CTGCGGCGAGCGGTAT		GTCGGTTGTCCTTGGTGATGCGGCAGTCCCCGT/	/GACCGAGCGCAGCGAGTCAGTG		
#1	Forward	CATGAAGCGGAAGGCACTATTCACCTGCCCCTTCA/	/GACCGAGCGCAGCGAGTCAGTG	0/0	GTCGGTTGTCCTTGGTGATGCGGCAGTCCCCGT/	/GTTCGGCTGCGGCGAGGAGTATCAGCTCACTCAAAG	0/0	negative
#5	Reverse	CATGAAGCGGAAGGCACTATTCACCTGCCCCTTCA/	/GTTCGGCTGCGGCGAGCGGTAT	0/0	GTCGGTTGTCCTTGGTGATGCGGCAGTCCCCGT/	/GACCGAGCGCAGCGAGTCAGTG	0/0	negative
#9	Reverse	CATGAAGCGGAAGGCACTATTCACCTGCCCCTTCA/	/GTTCGGCTGCGGCGAGCGGTAT	0/0	GTCGGTTGTCCTTGGTGATGCGGCAGTCCCCGT/	/GACCGAGCGCAGCGAGTCAGTG	0/0	negative
#12	Forward	CATGAAGCGGAAGGCACTATTCACCTGCCCCTTCA/	/GACCGAGCGCAGCGAGTCAGTG	0/0	GTCGGTTGTCCTTGGTGATGC------------/	/GTTCGGCTGCGGCGAGGAGTATCAGCTCACTCAAAG	-12/0	negative
#14	Forward	CATGAAGCGGAAGGCACTATTCACCTGCCCCTTCA/	/GACCGAGCGCAGCGAGTCAGTG	0/0	GTCGGTTGTCCTTGGTGATGCGGCAGTCCCCGT/	/GTTCGGCTGCGGCGAGGAGTATCAGCTCACTCAAAG	0/0	negative
#15	Reverse	CATGAAGCGGAAGGCACTATTCACCTGCCCCTTCA/	/GTTCGGCTGCGGCGAGCGGTAT	0/0	GTCGGTTGTCCTTGGTGATGCGGCAGTCCCCGT/	/GACCGAGCGCAGCGAGTCAGTG	0/0	negative
#16	Reverse	CATGAAGCGGAAGGCACTATTCACCTGCCCCTTCA/	/GTTCGGCTGCGGCGAGCGGTAT	0/0	GTCGGTTGTCCTTGGTGATGCGGCAGTCCCC--/	/GACCGAGCGCAGCGAGTCAGTG	-2/0	negative
#17	Reverse	CATGAAGCGGAAGGCACTATTCACCTGCCCCTTCA/	/GTTCGGCTGCGGCGAGCGGTAT	0/0	GTCGGTTGTCCTTGGTGATGCGGCAGTCC----/	/----------AGCGAGTCAGTG	-4/-10	negative
#21	Forward	CATGAAGCGGAAGGCACTATTCACCTGCCCCTTCA/	/GACCGAGCGCAGCGAGTCAGTG	0/0	GTCGGTTGTCCTTGGTGATGCGGCAGTCCCCGT/	/GTTCGGCTGCGGCGAGGAGTATCAGCTCACTCAAAG	0/0	negative
#26	Forward	CATGAAGCGGAAGGCACTATTCACCTGCCCCTTCA/	/GACCGAGCGCAGCGAGTCAGTG	0/0	GTCGGTTGTCCTTGGTGATGCGGCAGTCCCCGT/	/GTTCGGCTGCGGCGAGGAGTATCAGCTCACTCAAAG	0/0	negative
#29	Reverse	CATGAAGCGGAAGGCACTATTCACCTGCCCCTTCA/	/GTTCGGCTGCGGCGAGCGGTAT	0/0	GTCGGTTGTCCTTGGTGATGCGGCAGTCCCCGT/	/GACCGAGCGCAGCGAGTCAGTG	0/0	negative
#30	Forward	CATGAAGCGGAAGGCACTATTCACCTGCCCCTTCA/	/GACCGAGCGCAGCGAGTCAGTG	0/0	GTCGGTTGTCCTTGGTGATGCGGCAGTCCCCGT/	/GTTCGGCTGCGGCGAGGAGTATCAGCTCACTCAAAG	0/0	negative
#33	Forward	CATGAAGCGGAAGGCACTATTCACCTGCCCCTTCA/	/GACCGAGCGCAGCGAGTCAGTG	0/0	GTCGGTTGTC-----------------------/	/-----------------------AGCTCACTCAAAG	-23/-23	negative
#35	Forward	CATGAAGCGGAAGGCACTATTCACCTGCCCCTTCA/	/GACCGAGCGCAGCGAGTCAGTG	0/0	GTCGGTTGTCCTTGGTGATGCGG----------/	/GGTTCGGCTGCGGCGAGGAGTATCAGCTCACTCAAAG	-10/+1	negative
#38	Reverse	CATGAAGCGGAAGGCACTATTCACCTGCCCCTTCA/A	/GTTCGGCTGCGGCGAGCGGTAT	+1/0	GTCGGTTGTCCTTGGTGATGCGGCAGTCCCCGT/	/GACCGAGCGCAGCGAGTCAGTG	0/0	negative
#40	Forward	CATGAAGCGGAAGGCACTATTCACCTGCCCCTTCA/	/GACCGAGCGCAGCGAGTCAGTG	0/0	GTCGGTTGTCCTTGGTGATGCGGCAGTCCCC--/	/GTTCGGCTGCGGCGAGGAGTATCAGCTCACTCAAAG	-2/0	negative
#41	Forward	CATGAAGCGGAAGGCACTATTCACCTGCCCCTTCA/	/GACCGAGCGCAGCGAGTCAGTG	0/0	GTCGGT---------------------------/	/GTTCGGCTGCGGCGAGGAGTATCAGCTCACTCAAAG	-27/0	negative
#44	Forward	CATGAAGCGGAAGGCACTATTCACCTGCCCCTTCA/	/GACCGAGCGCAGCGAGTCAGTG	0/0	GTCGGTTGTCCTTGGTGATGCGGCAGTCCCCGT/	/GTTCGGCTGCGGCGAGGAGTATCAGCTCACTCAAAG	0/0	negative

Supplementary Table S3 Direct sequencing of additional knock-in clones

The sequences of knock-in clones in addition to the supplementary fig.2, which were analyzed by direct sequencing, were shown. The predicted sequences with forward and reverse direction were shown in colored sequences.
PAM sequences were shown with red and bold. The indel were colored with red. The predicted sequeces show the sequences with precise knocked-in (see also Supplementary Fig. S2).

Clone	Upstream (VDR genomeDNA)	Upstream (Plasmid DNA)	indel size	Downstream (VDR genomeDNA)	Downstream (Plasmid DNA)	indel size	Amplicon 6
**Predicted sequence (Forward)**	CATGAAGCGGAAGGCACTATTCACCTGCC**CCT**TCA/	/GACCGAGCGCAGCGAGTCAGTG		GTCGGTTGTCCTTGGTGATGCGGCAGTCCCCGT/	/GTT**CGG**CTGCGGCGAGCGGTATCAGCTCACTCAAAG		
**Predicted sequence(Reverse)**	CATGAAGCGGAAGGCACTATTCACCTGCC**CCT**TCA/	/GTT**CGG**CTGCGGCGAGCGGTAT		GTCGGTTGTCCTTGGTGATGCGGCAGTCCCCGT/	/GACCGAGCGCAGCGAGTCAGTG		
#2	CATGAAGCGGAAGGCACTATTCACCTGCCCCTTCA/	/GACCGAGCGCAGCGAGTCAGTG	0/0	GTCGGTTGTCCTTGGTGATGCGGCAGTCCCCGT/	/GACCGAGCGCAGCGAGTCAGTG	0/0	negative
#3	n. d.	n. d.		n. d.	n. d.		positive
#4	n. d.	n. d.		n. d.	n. d.		negative
#6	n. d.	n. d.		n. d.	n. d.		positive
#7	n. d.	n. d.		n. d.	n. d.		positive
#8	n. d.	n. d.		n. d.	n. d.		negative
#10	CATGAAGCGGAAGGCACTATTCACCTGCCCCTTCA/	/GACCGAGCGCAGCGAGTCAGTG	0/0	GTCGGTTGTCCTTGGTGATGCGGCAGTCCCCGT/	/GTTCGGCTGCGGCGAGGAGTATCAGCTCACTCAAAG	0/0	negative
#11	n. d.	n. d.		n. d.	n. d.		positive
#13	n. d.	n. d.		n. d.	n. d.		positive
#18	n. d.	n. d.		n. d.	n. d.		positive
#19	CATGAAGCGGAAGGCACTATTCACCTGCCCCTTCA/	/GACCGAGCGCAGCGAGTCAGTG	0/0	GTCGGTTGTCCTTGGTGATGCGGCAGTCCCCGT/	/GTTCGGCTGCGGCGAGGAGTATCAGCTCACTCAAAG	0/0	negative
#20	n. d.	n. d.		n. d.	n. d.		positive
#22	n. d.	n. d.		n. d.	n. d.		negative
#23	n. d.	n. d.		n. d.	n. d.		negative
#24	CATGAAGCGGAAGGCACTATTCACCTGCCCCTTCA/	/GACCGAGCGCAGCGAGTCAGTG	0/0	GTCGGTTGTCCTTGGTGATGCGGCAGTCCCCGT/	/GACCGAGCGCAGCGAGTCAGTG	0/0	negative
#25	n. d.	n. d.		n. d.	n. d.		positive
#27	n. d.	n. d.		n. d.	n. d.		positive
#28	n. d.	n. d.		n. d.	n. d.		negative
#31	CATGAAGCGGAAGGCACTATTCACCTGCCCCTTCA/A	/GTTCGGCTGCGGCGAGCGGTAT	+1/0	GTCGG--/	/--CAAAG	-42/-46	negative
#32	n. d.	n. d.		n. d.	n. d.		positive
#34	n. d.	n. d.		n. d.	n. d.		negative
#36	n. d.	n. d.		n. d.	n. d.		negative
#37	CATGAAGCGGAAGGCACTATTCACCTGCCCCTTCA/A	/GTTCGGCTGCGGCGAGCGGTAT	+1/0	GTCGGTTGTCCTTGGTGATGCGGCAGTCCCCGT/	/GACCGAGCGCAGCGAGTCAGTG	0/0	negative
#39	n. d.	n. d.		n. d.	n. d.		positive
#42	n. d.	n. d.		n. d.	n. d.		negative
#43	n. d.	n. d.		n. d.	n. d.		positive
#45	CATGAAGCGGAAGGCACT------------------------ /	/--(412bp)--CTCGCGTTAACGCTAGGATGGATGTTTT	-17/-412	n. d.	n. d.		negative
#46	n. d.	n. d.		n. d.	n. d.		negative
#47	n. d.	n. d.		n. d.	n. d.		negative
#48	n. d.	n. d.		n. d.	n. d.		negative
#49	CATGAAGCGGAAGGCACTATTCACCTGCCCCTTCA/A	/GTTCGGCTGCGGCGAGCGGTAT	+1/0	GTCGGTTGTCCTTGGTGATGCGGCAGTCCCCGT/	/GACCGAGCGCAGCGAGTCAGTG	0/0	negative
#50	n. d.	n. d.		n. d.	n. d.		positive
#51	n. d.	n. d.		n. d.	n. d.		positive
#52	CATGAAGCGGAAGGCACTATTCACCTGCCCCTTCA/	/Cas9 sequence (AAGCACGAGCGGCACCCCATCTT…)	0/Cas9	GTCGGTTGTCCTTGGTGATGCGGCAGTCCCCGT/	/GTTCGGCTGCGGCGAGGAGTATCAGCTCACTCAAAG	0/0	negative
#53	n. d.	n. d.		n. d.	n. d.		positive
#54	n. d.	n. d.		n. d.	n. d.		positive
#55	CATGAAGCGGAAGGCACTATTCACCTGCCCCTTCA/	/GACCGAGCGCAGCGAGTCAGTG	0/0	n. d.	n. d.		negative
#56	CATGAAGCGGAAGGCACTATTCACCTGCCCCTTCA/	/GTTCGGCTGCGGCGAGCGGTAT	0/0	GTCGGTTGTCCTTGGTGATGCGGCAGTCCCCGT/	/GACCGAGCGCAGCGAGTCAGTG	0/0	negative
#57	CATGAAGCGGAAGGCACTATTCACCTGCCCCTTCA/	/GTTCGGCTGCGGCGAGCGGTAT	0/0	GTCGGTTGTCCTTGGTGATGCGGCAGTCCCCGT/	/GACCGAGCGCAGCGAGTCAGTG	0/0	negative
#58	CATGAAGCGGAAGGCACTATTCACCTGCCCCTTCA/A	/GTTCGGCTGCGGCGAGCGGTAT	+1/0	GTCGGTTGTCCTTGGTGATGCGGCAGTCCCCGT/	/GACCGAGCGCAGCGAGTCAGTG	0/0	negative
#59	CATGAAGCGGAAGGCACTATTCACCTGCCCCTTCA/	/GTTCGGCTGCGGCGAGCGGTAT	0/0	GTCGGTTGTCCTTGGTGATGCGGCAGTCCCCGT/	/GACCGAGCGCAGCGAGTCAGTG	0/0	negative
#60	n. d.	n. d.		n. d.	n. d.		positive
#61	CATGAAGCGGAAGGCACTATTCACCTGCCCCTTCA/	/GTTCGGCTGCGGCGAGCGGTAT	0/0	GTCGGTTGTCCTTGGTGATGCGGCAGTCCCCGT/	/GACCGAGCGCAGCGAGTCAGTG	0/0	negative
#62	CATGAAGCGGAAGGCACTATTCACCTGCCCCTTCA/	/GTTCGGCTGCGGCGAGCGGTAT	0/0	GTCGGTTGTCCTTGGTGATGCGGCAGTCCCCGT/	/GACCGAGCGCAGCGAGTCAGTG	0/0	negative
#63	CATGAAGCGGAAGGCACTATTCACCTGCCCCTTCA/	/GTTCGGCTGCGGCGAGCGGTAT	0/0	GTCGGTTGTCCTTGGTGATGCGGCAGTCCCCGT/	/GACCGAGCGCAGCGAGTCAGTG	0/0	negative
#64	CATGAAGCGGAAGGCACTATTCACCTGCCCCTTCA/	/GTTCGGCTGCGGCGAGCGGTAT	0/0	GTCGGTTGTCCTTGGTGATGCGGCAGTCCCCGT/	/GACCGAGCGCAGCGAGTCAGTG	0/0	negative
#65	CATGAAGCGGAAGGCACTATTCACCTGCCCCTTCA/	/GACCGAGCGCAGCGAGTCAGTG	0/0	GTCGGTTGTCCTTGGT-----------------/	/GTTCGGCTGCGGCGAGGAGTATCAGCTCACTCAAAG	-17/0 (1nt overlapped)	negative
#66	CATGAAGCGGAAGGCACTATTCACCTGCCCCTTCA/	/GACCGAGCGCAGCGAGTCAGTG	0/0	GTCGGTTGTCCTTGGTGATGCGGCAGTCCCCGT/	/GTTCGGCTGCGGCGAGGAGTATCAGCTCACTCAAAG	0/0	negative
#67	CATGAAGCGGAAGGCACTATTCACCTGCCCCTTCA/	/GTTCGGCTGCGGCGAGCGGTAT	0/0	GTCGGTTGTCCTTGGTGATGCGGCAGTCCCCGT/	/GACCGAGCGCAGCGAGTCAGTG	0/0	negative
#68	CATGA------------------------------/	/GTTCGGCTGCGGCGAGCGGTAT	-30/0	GTCGGTTGTCCTTGGTGATGCGGCAGTCCCC--/	/GACCGAGCGCAGCGAGTCAGTG	-2/0 (1nt overlapped)	negative
#69	CATGAAGCGGAAGGCACTATTCACCTGCCCCTTCA/	/GTTCGGCTGCGGCGAGCGGTAT	0/0	GTCGGTTGTCCTTGGTGATGCGGCAGTCCCCGT/	/GACCGAGCGCAGCGAGTCAGTG	0/0	negative
#70	CATGAAGCGGAAGGCACTATTCACCTGCCCCTTCA/	/GACCGAGCGCAGCGAGTCAGTG	0/0	GTCGGTTGTCCTTGGTGATGCGGCAGTCCCCGT/	/GTTCGGCTGCGGCGAGGAGTATCAGCTCACTCAAAG	0/0	negative

Supplmentary Table S4 Summary of sequencing analysis of *VDR* alleles and functional assays of the *VDR* gene

Column1: clone number in which knock-in event was confirmed. Column2: the sequence detected by direct sequencing analysis of *VDR* gene in the knock-in clones

Column3-5: Summary of the results shown in Fig. 4

Clone	Indel mutations	VDR protein expression	*CYP24A1* induction	Reporter responsivility
#1	delta 2bp	undetectable	no induction	nonresponsive
#5	delta 2bp, +1bp	undetectable	no induction	nonresponsive
#9	delta 1bp, +1bp			
#12	delta 2bp, delta 3bp			
#14	delta 1bp, delta 2bp			
#15	delta 1bp			
#16	delta 2 bp, Wild-type			
#17	+1bp			
#21	delta 1bp, +1bp			
#26	delta 13bp, delta 10bp, +4bp			
#29	delta 1bp, +1bp	undetectable	no induction	nonresponsive
#30	delta 1bp, delta 2bp			
#33	delta 120bp, delta 1bp, +1bp	undetectable	no induction	nonresponsive
#35	delta 1bp, delta 10bp	undetectable	no induction	
#38	delta 4bp			
#40	delta 1bp, delta 2bp	undetectable	no induction	
#41	delta 3bp			
#44	delta 1bp, delta 3bp			

Supplmentary Table S5 Primers used in this study

Name	Sequence	Construct name	Backbone plasmid
Puro	5'-GTGGCGCGCCGGCCGCGAAGGATCTG-3'<br>5'-ACTTAATTAAATAAGATACATTGATGAG-3'	pVKG1-Puro	pENTR
EGFP_pA	5'-ATTCTAGAGCTAGCGATGGTGAGCAAGGGCGAGG-3'<br>5'-ATTTAAATTCGAATT ATAAGATACATTGATGAGTT-3'	pGFP-RFPuro	pCDH
SV40promoter_BSD_pA	5'-CACCGGATCCGGCTGTGGAATGTG-3'<br>5'-CCAGACATGATAAGATACATTGATG-3'	pVKG1-BSD	pENTR
human_CYP24A1_PromoterS	5'-AAACTCGAGATTGTGCAAGCGCCGGCGGC-3'<br>5'-AAAGATATCATGTTCCTATGCCCAGGGAC-3'	pVDRE-Luc	pGL4.27
human_VDR	5'-AAAGAATTCATGGAGGCAATGGCGGCCAG-3'<br>5'-AAACTCGAGTCAGGAGATCTCATTGCCAAAC-3'	hVDR expression vector	pcDNA3FLAG

Name	Sequence	Primer name
Amplicon_1_for_VDR (Fig. 1d)	5'-TTCCTGTCTAGCGGTACGCG-3'<br>5'-GGTGGGCCTCATGTCTTCTG-3'	VKG1_250R<br>VDRgenome_del3F
Amplicon_2_for_VDR (Fig. 1d)	5'-GCCTATGGAAAAACGCCAGC-3'<br>5'-CCTTCATCATGCCGATGTCC-3'	VKG1_4561F<br>VDRgenome_del1R
Amplicon_3_for_VDR (Fig. 1d)	5'-GCCTATGGAAAAACGCCAGC-3'<br>5'-GGTGGGCCTCATGTCTTCTG-3'	VKG1_4561F<br>VDRgenome_del3F
Amplicon_4_for_VDR (Fig. 1d)	5'-TTCCTGTCTAGCGGTACGCG-3'<br>5'-TGCCCAAACTTGCAGGAGAG-3'	VKG1_250R<br>VDRgenome_del3R
Amplicon_5_for_VDR (Fig. 1d)	5'-ATGGCCGAGTTGAGCGGTTC-3'<br>5'-AGACCCTTGCCCTGGTGGTC-3'	PuroF<br>PuroR
Amplicon_6_for_VDR (Fig. 1d)	5'-GCCTATGGAAAAACGCCAGC-3'<br>5'-TTCCTGTCTAGCGGTACGCG-3'	VKG1_4561F<br>VKG1_250R
Amplicon_1_for_AAVS1 (Fig. 2d)	5'-GGAAATGGGGGTGTGTCACC-3'<br>5'-GGGTCTTGTAGTTGCCGTCG-3'	AAVS_F5<br>EGFP_R2
Amplicon_2_for_AAVS1 (Fig. 2d)	5'-CCCTACCCCCCTTACCTCTC-3'<br>5'-GCAGAGCTCGTTTAGTGAACCG-3'	AAVS_R6<br>EGFP_F4
Amplicon_3_for_AAVS1 (Fig. 2d)	5'-GGAAATGGGGGTGTGTCACC-3'<br>5'-AACCGTCAGATCGCCTGGAG-3'	AAVS_F5<br>EGFP_F2
Amplicon_4_for_AAVS1 (Fig. 2d)	5'-ACCTCTCTAGTCTGTGCTAGC-3'<br>5'-GGGTCTTGTAGTTGCCGTCG-3'	AAVS_R5<br>EGFP_R2
Amplicon_5_for_AAVS1 (Fig. 2d)	5'-AACCGTCAGATCGCCTGGAG-3'<br>5'-GGGTCTTGTAGTTGCCGTCG-3'	EGFP_F2<br>EGFP_R2
Amplicon_Puro (Fig. 2d)	5'-ATGGCCGAGTTGAGCGGTTC-3'<br>5'-AGACCCTTGCCCTGGTGGTC-3'	PuroF<br>PuroR
VDR cleaving site (Fig. 3d)	5'-GGTGGGCCTCATGTCTTCTG-3'<br>5'-TGCCCAAACTTGCAGGAGAG-3'	VDRgenome_del3F<br>VDRgenome_del3R
hCYP24A1_qPCR (Fig. 4b)	5'-CAGCGAACTGAACAAATGGTCG-3'<br>5'-GCTCGACTGGAGTGACCATC-3'	hCYP24A1_qPCR_For<br>hCYP24A1_qPCR_Rev
hACTB_qPCR (Fig. 4b)	5'-ATTGGCAATGAGCGGTTC-3'<br>5'-CGTGGATGCCACAGGACT-3'	hACTB_qPCR_For<br>hACTB_qPCR_Rev
Amplicon_BSD (Fig. S4a)	5'-CTGAAGACTACAGCGTCGCCC-3'<br>5'-TTCTCATTTCCGATCGCGACG-3'	BSD_F<br>BSD_R
Amplicon_1_for_HR (Fig. S5a)	5'-TTCCTGTCTAGCGGTACGCG-3'<br>5'-TGCACCAGAGGGATGGAGAG-3'	VKG1_250R<br>HRgenome_F2
Amplicon_2_for_HR (Fig. S5a)	5'-GCCTATGGAAAAACGCCAGC-3'<br>5'-TCATGCTGTCCCGAGTCCAC-3'	VKG1_4561F<br>HRgenome_R1
Amplicon_3_for_HR (Fig. S5a)	5'-GCCTATGGAAAAACGCCAGC-3'<br>5'-GCGATGGGAACCTTGGGTAC-3'	VKG1_4561F<br>HRgenome_F1
Amplicon_4_for_HR (Fig. S5a)	5'-TTCCTGTCTAGCGGTACGCG-3'<br>5'-TCATGCTGTCCCGAGTCCAC-3'	VKG1_250R<br>HRgenome_R1
Amplicon_5_for_HR (Fig. S5a)	5'-ATGGCCGAGTTGAGCGGTTC-3'<br>5'-AGACCCTTGCCCTGGTGGTC-3'	PuroF<br>PuroR
Amplicon_6_for_HR (Fig. S5a)	5'-GCCTATGGAAAAACGCCAGC-3'<br>5'-TTCCTGTCTAGCGGTACGCG-3'	VKG1_4561F<br>VKG1_250R

Supplymental information

Figure 1d

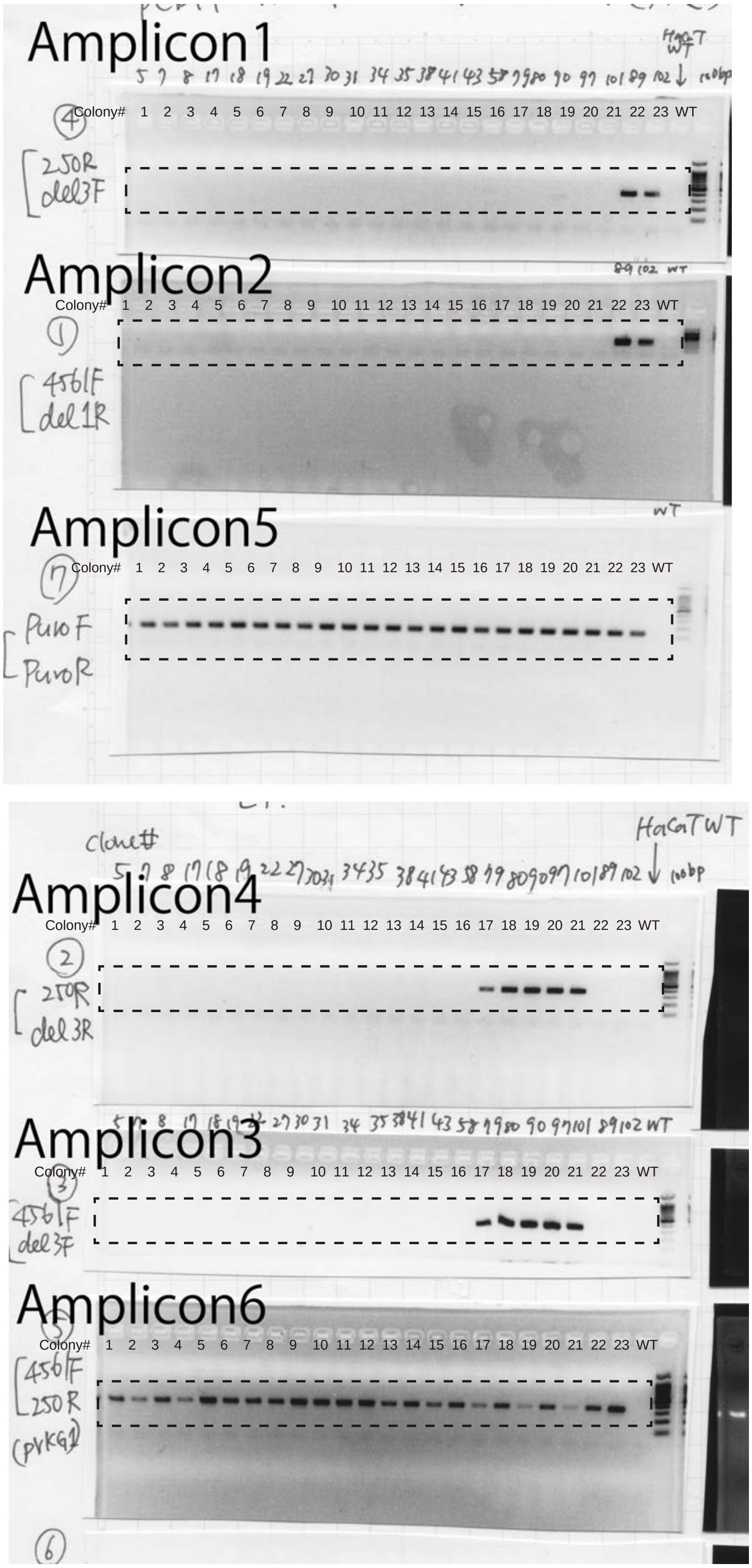

Supplymental information

Figure 2d

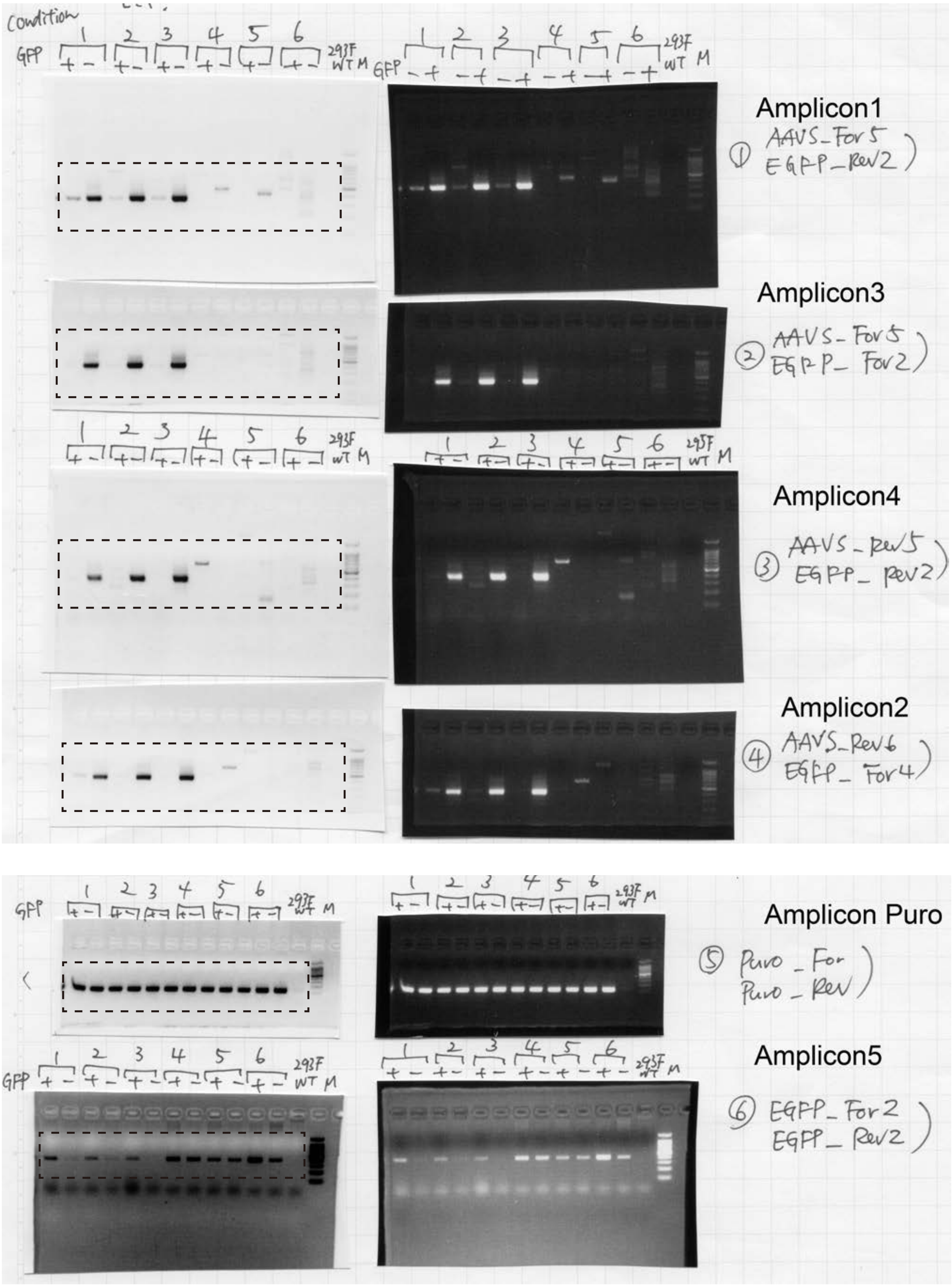

Supplymental information

Figure 3a

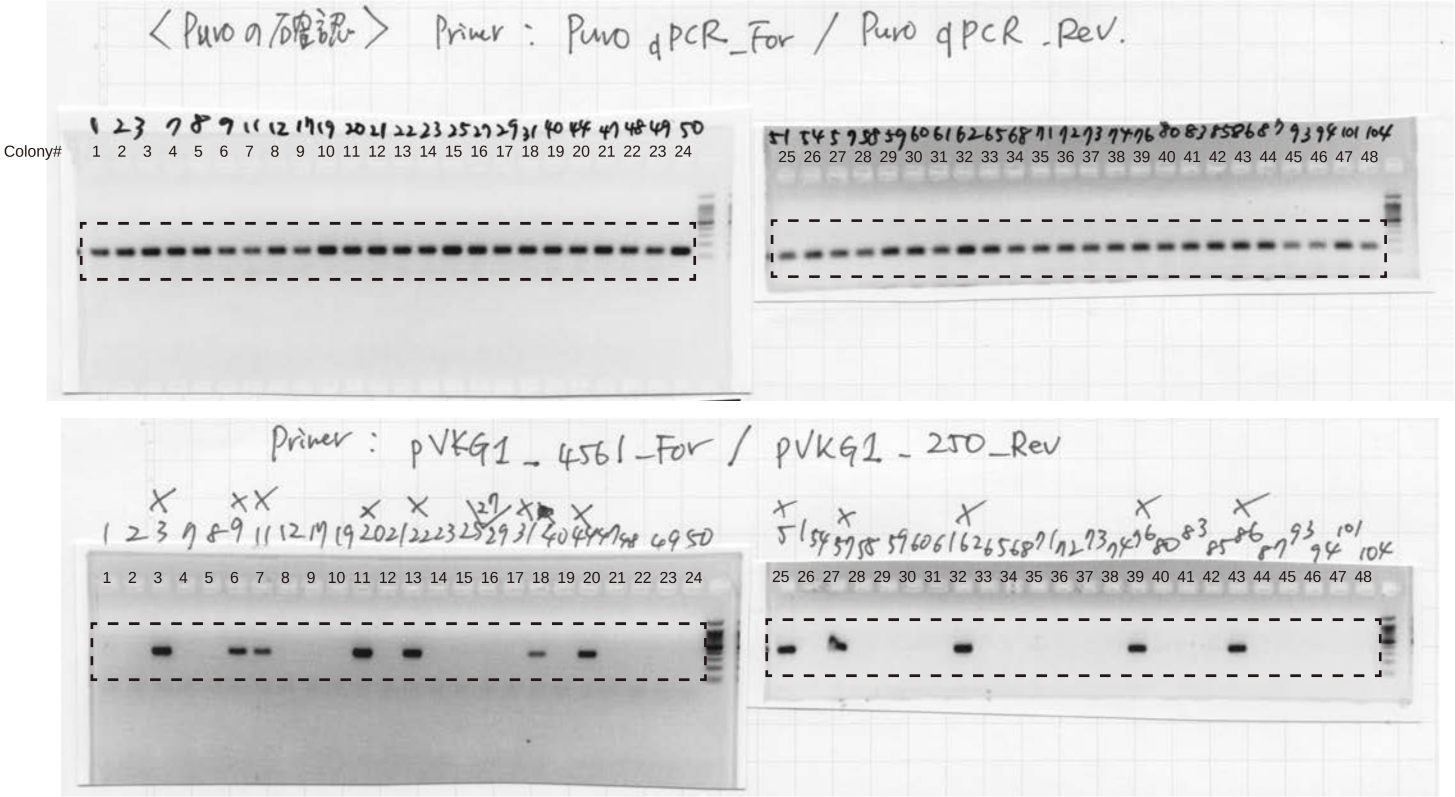

Supplymental information

Figure 3b

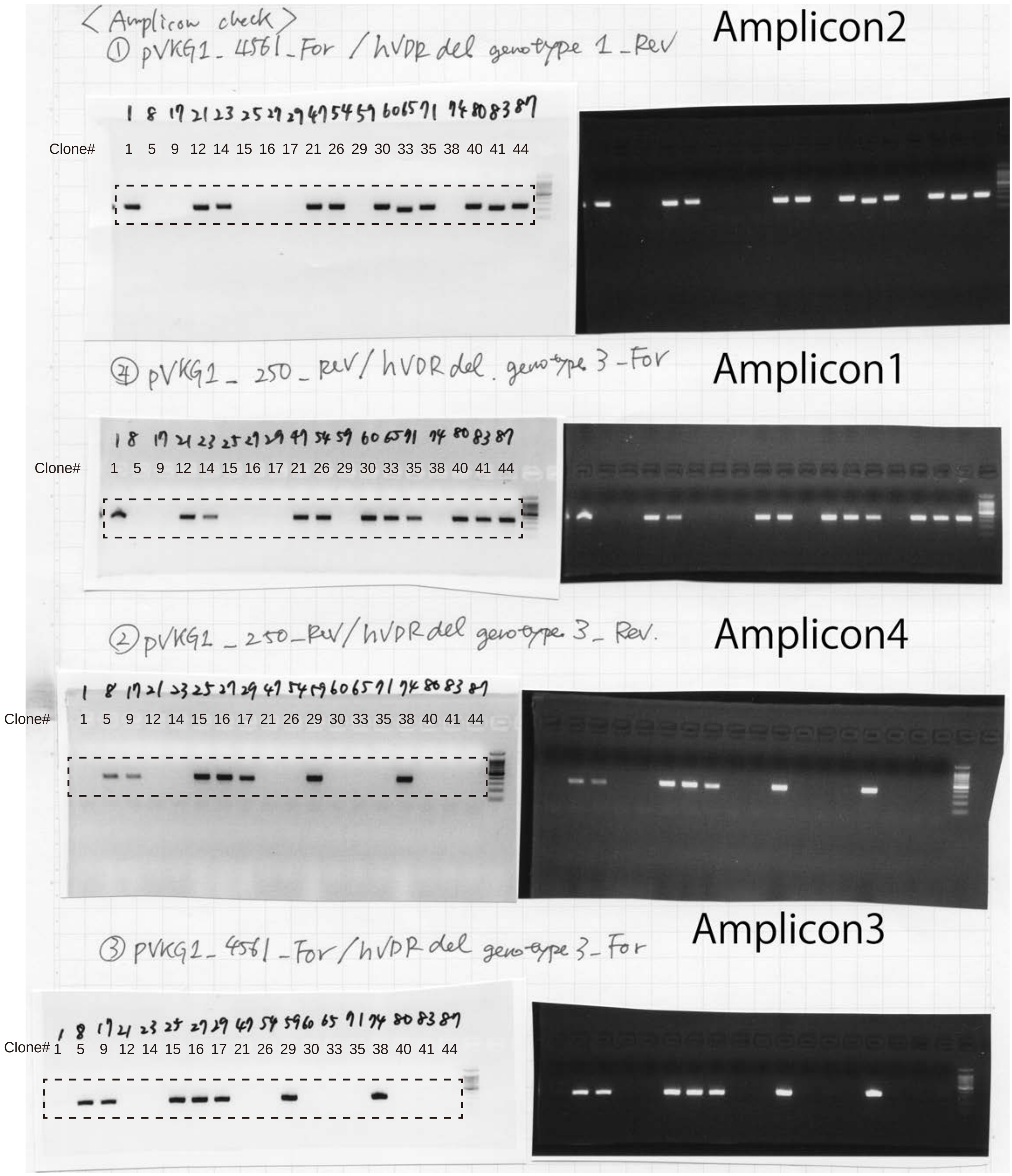

Supplymental information

Figure 4a

αVDR

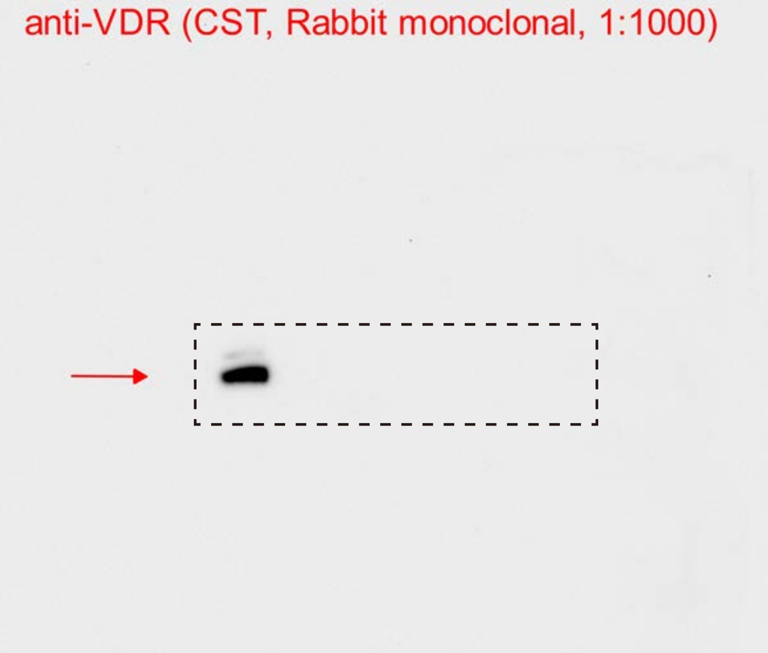

αVDR / Ladder marker

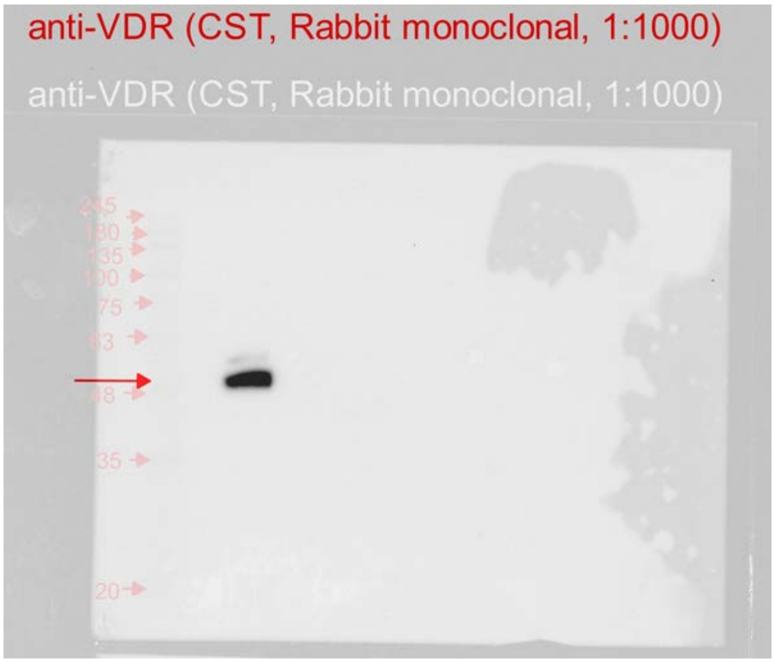

αHsp90

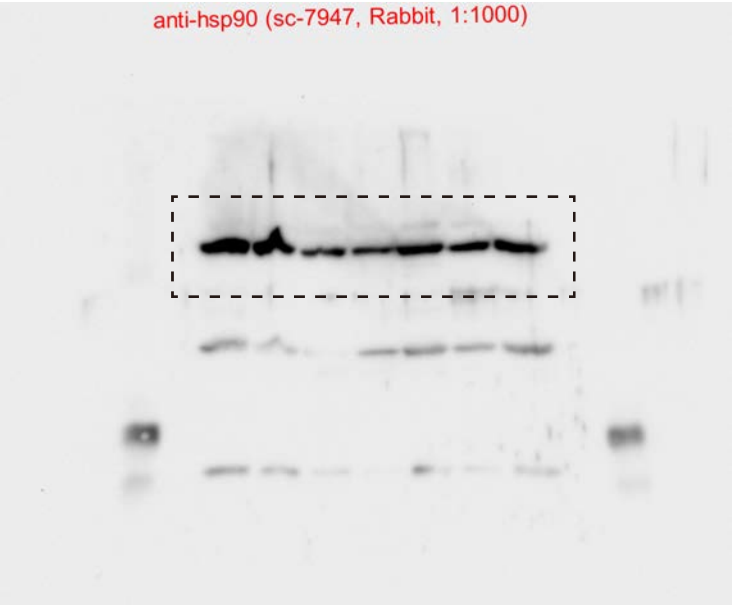

αHsp90 / Ladder marker

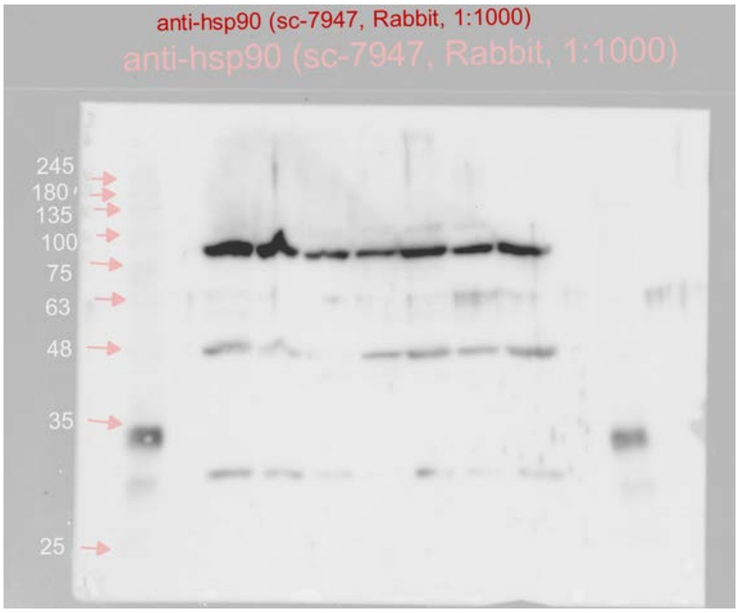

αβ-ACT

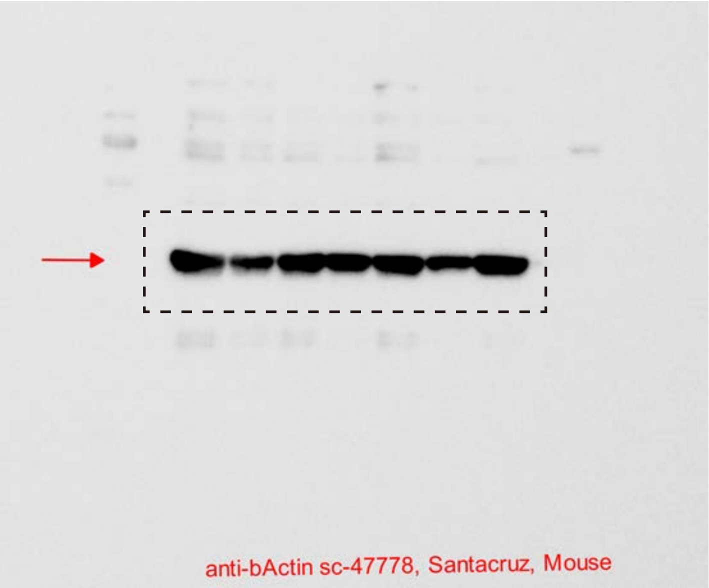

αβ-ACT / Ladder marker

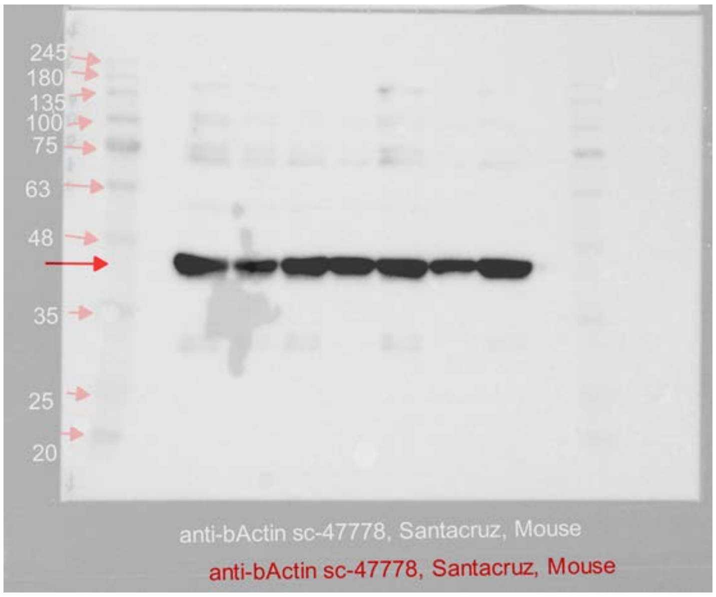

Supplymental information

Supplymentary Figure S4a

Amplicon 1

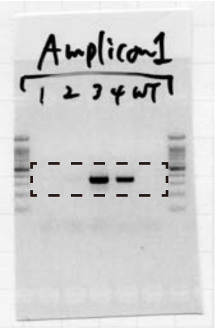

Amplicon 2

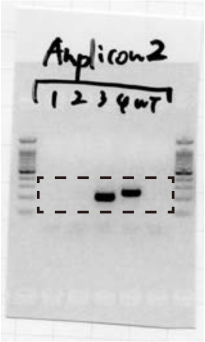

Amplicon 3

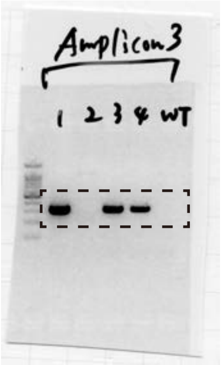

Amplicon 4

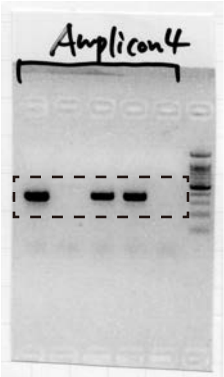

M : 100bp ladder marker

Amplicon Puro

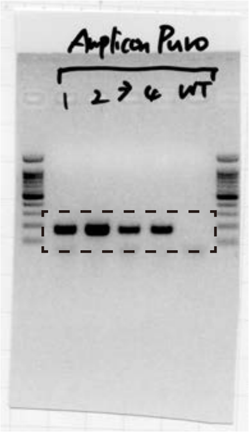

Amplicon BSD

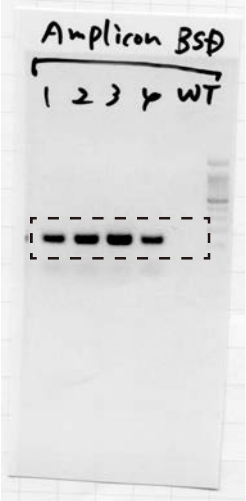

Amplicon 6

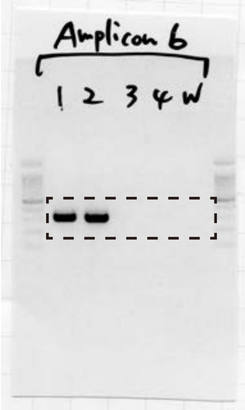

Supplymental information

Supplymentary Figure S4c

αVDR

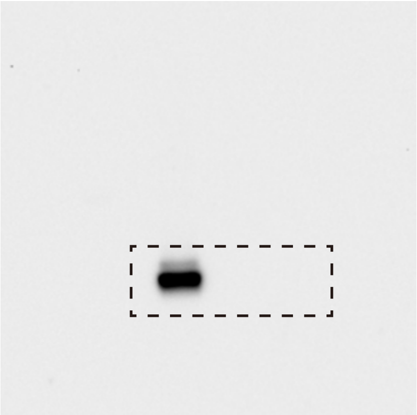

αVDR / Ladder marker

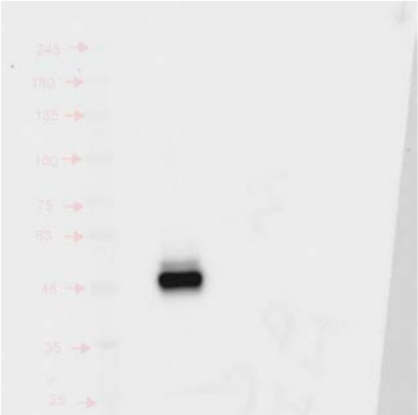

αHsp90

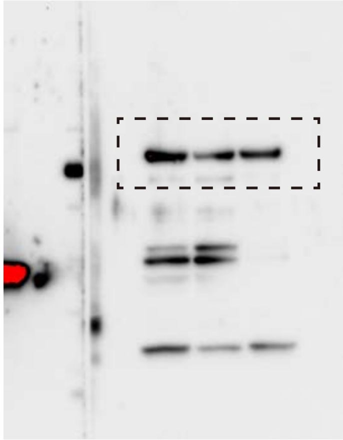

αHsp90 / Ladder marker

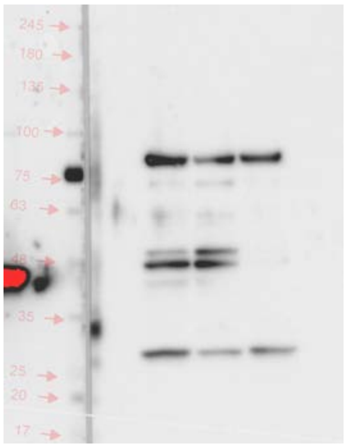

αβ-ACT

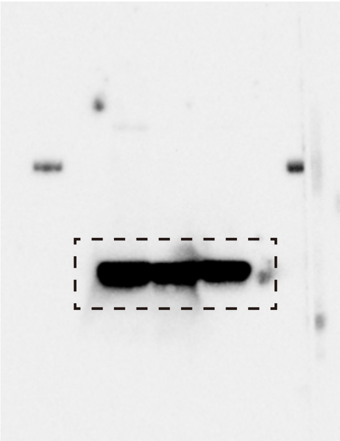

αβ-ACT / Ladder marker

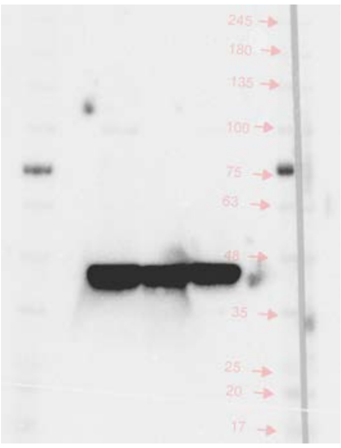

Supplymental information

Supplymentary Figure S5a

Amplicon 1

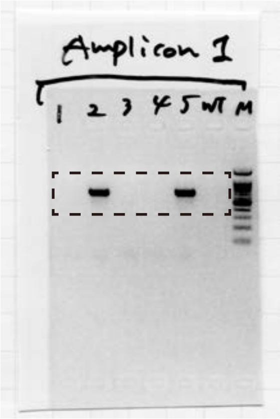

Amplicon 2

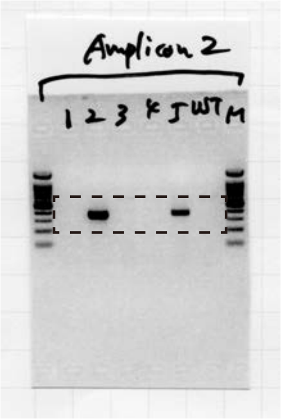

Amplicon 3

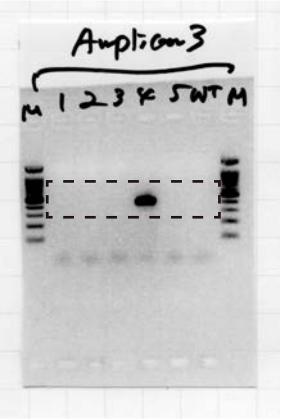

Amplicon 4

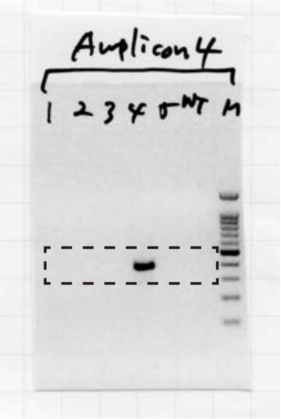

M : 100bp ladder marker

Amplicon 5

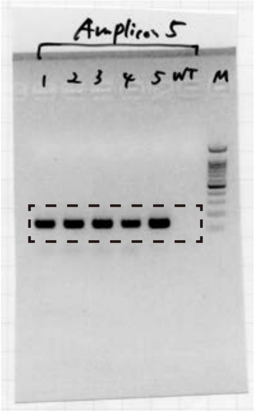

Amplicon 6

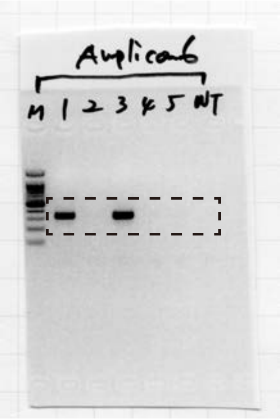

Supplymental information

Supplymentary Figure S5e

αHR

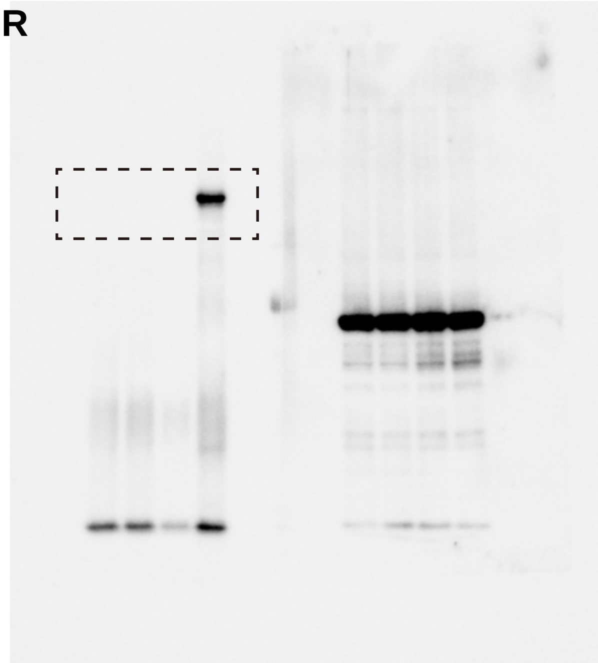

αHR / Ladder marker

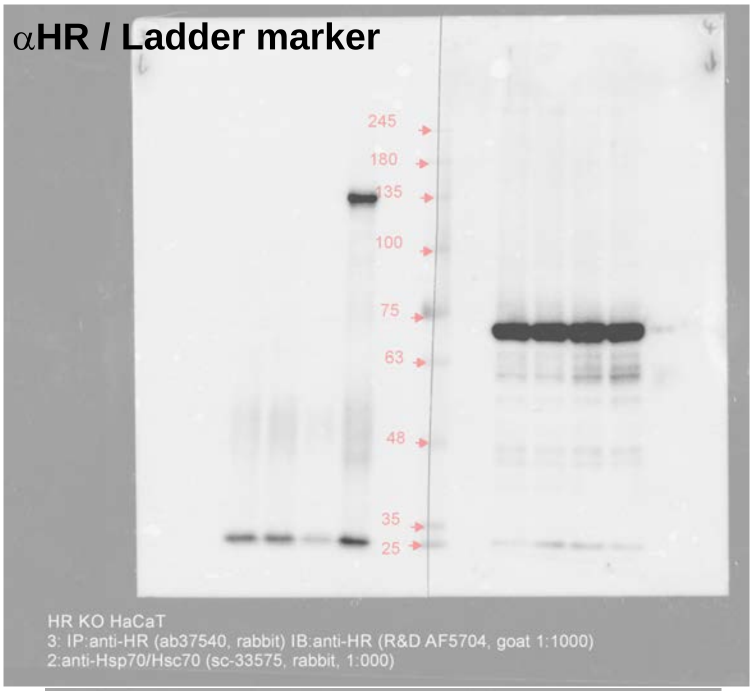

αHsp90

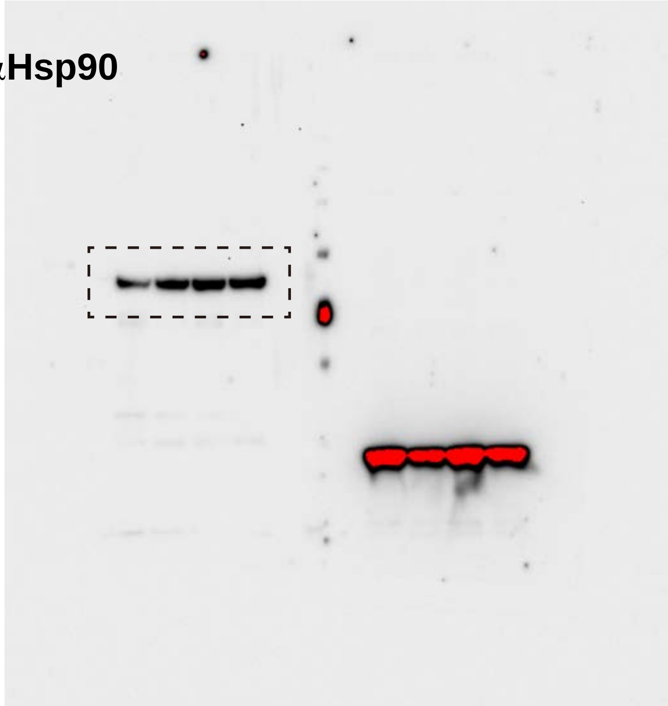

αHsp90 / Ladder marker

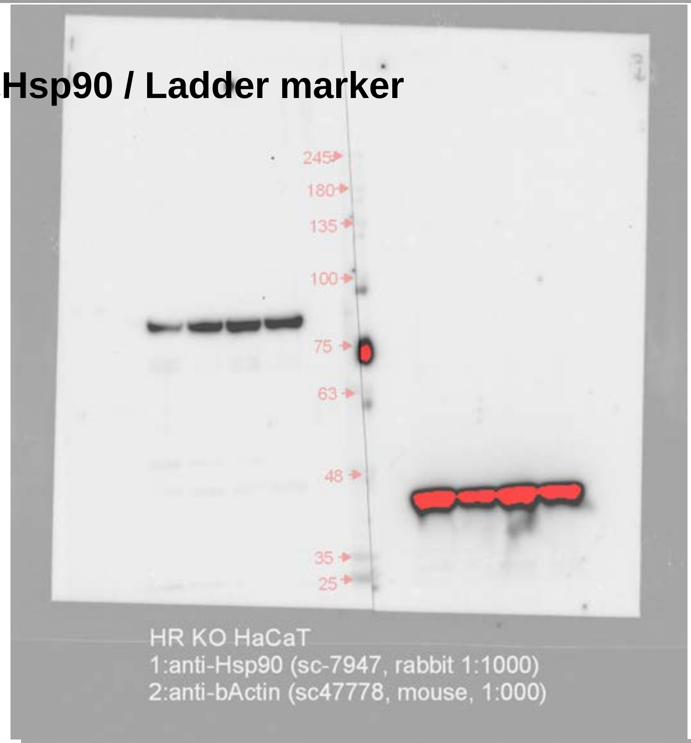

αβ-ACT

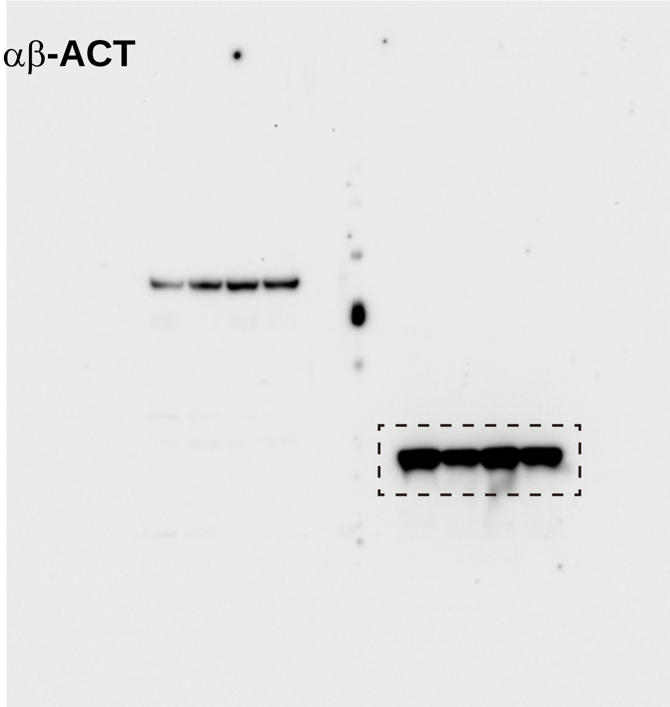

αβ-ACT / Ladder marker

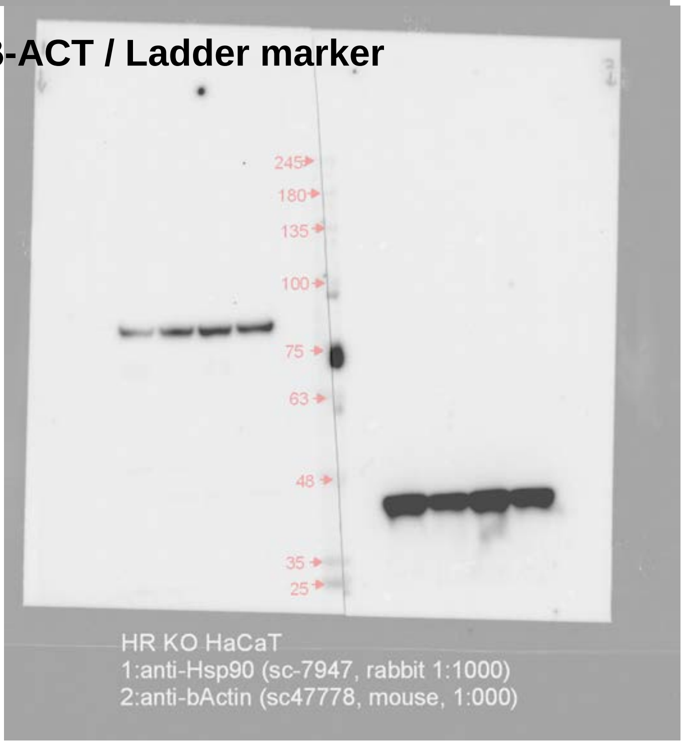

Supplement: Supplementary file 1 — Supplementary information [file 41598_2017_18911_MOESM1_ESM.pdf]
